# LncRNA *RP3-525N10.2-NFKB1-PROS1* triplet-mediated low *PROS1* expression is an onco-immunological biomarker in low-grade gliomas: a pan-cancer analysis with experimental verification

**DOI:** 10.1186/s12967-022-03536-y

**Published:** 2022-07-25

**Authors:** Yujie Zhou, Dongdong Xiao, Xiaobing Jiang

**Affiliations:** Department of Neurosurgery, Union Hospital, Tongji Medical College, Huazhong University of Science and Technology, 1277 Jiefang Avenue, Wuhan, 430022 China

**Keywords:** Carcinogenesis, Immunohistochemistry, Long noncoding rna, Transwell assays, Macrophages, Transcription factors, Tumor microenvironment

## Abstract

**Background:**

Glioma is the most common cancer in the central nervous system, and low grade gliomas are notorious for many types of tumors and heterogeneity. *PROS1* not only plays an important role in the blood coagulation system, and recent studies have found that it was correlated with the development of tumors, especially related to tumor immune infiltration. However, the study of underlying role and mechanism of PROS1 in gliomas, especially in low-grade gliomas, is almost absent.

**Methods:**

We integrated the information of patients with LGG in The Cancer Genome Atlas (TCGA) cohort and Chinese Glioma Genome Atlas (CGGA) cohort. Then, we systematically demonstrated the differences and prognostic prognosis value of *PROS1* based on multi-omics analyses. In addition, Cell counting kit-8 (CCK-8) assay, colony formation assay, 5-Ethynyl-2’-deoxyuridine (EdU) incorporation assay, and Transwell assays were performed to evaluate cell proliferation and invasion. qRT-PCR and immunohistochemistry were used to evaluate the expression of *PROS1* in LGG.

**Results:**

Various bioinformatics approaches revealed that *PROS1* was a valuable prognostic marker and may influence tumour development via distinct mechanisms, including expression of DNA methyltransferase, RNA modification, and DNA mismatch repair system genes, copy number variation, single nucleotide variation frequency, genomic heterogeneity, cancer stemness, DNA methylation, and alternative PROS1 splicing. Our analyses indicated that the long non-coding RNA RP3-525N10.2 may “decoy” or “guide” the transcription factor NFKB1 and prevent its association with PROS1, thereby reducing PROS1 expression and improving poor LGG prognosis. PROS1 expression was also closely associated with tumour infiltration by immune cells, especially tumour-associated macrophages, as well as the expression of various immune checkpoint inhibitors, immunomodulators, and immune cell markers.

**Conclusion:**

long non-coding RNA *RP3-525N10.2-NFKB1-PROS1* triplet-mediated *PROS1* expression could serve as a biomarker for cancer diagnosis, prognosis, therapy selection, and follow-up in LGG patients.

**Supplementary Information:**

The online version contains supplementary material available at 10.1186/s12967-022-03536-y.

## Introduction

Glioma is the most common malignancy in the brain. Low-grade gliomas (LGGs, grade II and III), originating from neuroepithelial tissue [1], have high mortality in patients and are difficult to diagnose due to high intratumoural heterogeneity leading to distinct biological behaviour. To date, the standard treatment for LGG is maximal safe resection with adjuvant radiochemotherapy. Despite improvements in LGG treatment, more than half of the LGGs progress to therapy-resistant high-grade aggressive gliomas [2]. Several studies examined O-6-methylguanine-DNA methyltransferase (*MGMT*) methylation, codeletion of chromosome arms 1p and 19q, and isocitrate dehydrogenase (*IDH*) mutations to provide insight into LGG pathogenesis and advance cancer therapies for patients with LGGs [3, 4]. However, these most widely utilised molecular biomarkers cannot adequately reflect individual heterogeneity and provide clinical stratification of LGG risk. Therefore, there is an urgent need to elucidate potential mechanisms of LGG progression, establish new drug targets, and identify effective biomarkers for patients at high risk of developing LGGs. Protein S1 (PROS1) is a well-known ligand of the TYRO3, AXL, and MERTK family of receptor tyrosine kinases. The duplication events that gave rise to these kinases may have occurred in the early metazoan evolution, about 6 million years ago [5]. PROS1 is a secreted water-soluble vitamin K-dependent protein that is γ-carboxylated within the N-terminal of the Gla domain. The Gla domain confers the ability of PROS1 to bind phosphatidylserine on the surface of apoptotic cells, and the C-terminal sex hormone-binding globulin-like module can bind and activate TYRO3 and MERTK [6]. PROS1 is a key plasma protein and plays critical roles in anticoagulation and the phagocytosis of apoptotic cells [7]. Tumour-secreted PROS1 can decrease the expression of macrophage M1 cytokines in vitro and in vivo [8]. PROS1 plays an important role in inflammatory diseases, including periodontitis and glomerular injury [9, 10]. Moreover, *PROS1* has been identified as a potential target gene in several types of human cancers, including papillary thyroid carcinoma, oral squamous cell carcinoma, malignant thyroid cancer, intrahepatic cholangiocellular carcinoma, and glioblastoma [11–16]. However, a comprehensive study on *PROS1* expression, its prognostic value, and the underlying mechanisms in gliomas, especially in LGGs, is still missing. Additionally, the correlations of *PROS1* expression with multi-omic data and tumour infiltration by immune cells in LGG remain undetermined.

## Materials and methods

### Data download, process, and analysis

*PROS1* gene expression data were downloaded from the GTEx portal (https://gtexport.org/home/), the Cancer Cell Line Encyclopedia database (https://portals.broadinstitute.org/ccle/about), the TCGA database (https://genome-cancer.ucsc.edu/), and the CGGA database (http://www.cgga.org.cn/). These data were normalized, and differential expression analyses were performed for *PROS1* using the R package “limma” [28].

### Sangerbox tools

The free data analysis platform Sangerbox (http://www.sangerbox.com/tool) was used to validate the pan-cancer expression of *PROS1* and explore the correlation of *PROS1* expression with the expression of DNA methyltransferases, RNA modification genes, and DNA mismatch repair system genes, as well as copy number variation, single nucleotide variation frequency, genomic heterogeneity, and cancer stemness using Spearman’s or Pearson’s method.

### Gene expression profiling interactive analysis (GEPIA)

GEPIA (http://gepia.cancer-pku.cn/index.html) is a free web tool based on TCGA and GTEx data. In the current study, *PROS1* expression, survival analysis, and possible involvements of lncRNAs and TFs were evaluated using the GEPIA modules “Expression DIY” and “Survival”. In addition, the relationships between PROS1 and Gene markers were determined using Spearman’s correlation coefficient in the module “Correlation analysis”.

### Tumor immune estimation resource (TIMER)

TIMER (https://cistrome.shinyapps.io/timer/) is a web server for the comprehensive analysis of tumour-infiltrating immune cells. In our study, *PROS1* expression and survival were evaluated using the “Gene” and “Survival” modules. TIMER was also applied to investigate the relationships between *PROS1* expression and different gene marker sets of immune cells using the “Correlation” module [29].

### Tissue sampling from glioma patients

Fresh glioma tissues from histologically confirmed cases were obtained from the Union Hospital, Tongji Medical College, Huazhong University of Science and Technology. The study was approved by the Ethics Committee of the Union Hospital, Tongji Medical College, Huazhong University of Science and Technology.

### Cell culture, real-time PCR, and immunohistochemistry

Since there are no specific LGG cell lines, common glioma cell lines (U87, U251, and T98G) and the human astrocyte cell line NHA were used. Cells were cultured in Dulbecco’s modified Eagle’s medium (Gibco) containing 10% heat-inactivated foetal bovine serum and 1% penicillin/streptomycin. Real-time PCR was conducted to compare gene expression in 30 tumour samples with that in adjacent normal tissue. Real-time PCR was performed in triplicate using samples derived from three independent experiments. Primers for *PROS1* (forward, 5’-GTGCCTTCCCTTGAACCRRG-3’, reverse, 5’-CCACGCTGAGTGATCGATAGA-3’) and *GAPDH* (forward, 5’-AAAAGCATCACCCGGAGGAGAA-3’, reverse, 5’-AAGGAAATGAATGGGCAGCCG-3’) were used for qPCRs. Ten formalin-fixed, paraffin-embedded LGG tissues and normal brain tissues were used for immunohistochemistry stainings.

### lentivirus infection assay

Short hairpin RNA (shRNA) against PROS1 (shPROS1) and a negative control shRNA (sh NC) were designed and synthesised by genomeditech (Shanghai, China). In addition, the pcDNA3.1 vector (Vigene Biology) containing the full-length cDNA sequence of PROS1 was used to overexpress PROS1. The empty pcDNA3.1 vector was used as a negative control. The lentivirus pLent-shPROS1-GFP-Puro or its negative control (NC) pLent-GFP-Puro (genomeditech) was used to infect GBM cells with enhanced infection solution (genomeditech) according to the manufacturer’s protocol. Similarly, pLent-PROS1-GFP-Puro lentivirus or empty vector (vector) pLent-GFP-Puro lentivirus (genomeditech) was used to overexpress genes. Seventy two hours after the cells were infected with lentivirus, 2 μg/ml puromycin was added to kill the cells that had not been transfected.

### Cell counting Kit-8 assay

U87 and U251 cells was assessed with the Cell Counting Kit-8 (Dojindo Molecular Technologies, Kyushu, Japan) reagent according to the manufacturer’s instructions. Cells were inoculated on 96- well plates at a density of 1000 cells per well with 100 μl of medium. Every 24 h for a total of 96 h, CCK8 solution (10 μl) was added to each well, and the cells were further incubated at 37 °C for 3 h. The absorbance of each well was measured at 450 nm with a spectrophotometer.

### Colony formation assay

U87 and U251 Cells were prepared into a single-cell suspension, respectively, and seeded into a six-well plate (200 cells/well) for two-week incubation to form colonies. After staining with 0.01% crystal violet (Sigma), the colonies were subjected to microscopic examination. The rate of colony formation and survival fraction were calculated.

### Cell invasion assays

2 × 10^4^ cells were added into Matrigel-coated upper Transwell chambers for the invasion assay. The lower chambers were filled with DMEM containing 10% FBS. After incubation at 37 °C for 24 h, cells on the lower surface of the membrane were fixed in 100% methanol and stained with 0.1% crystal violet dye for 20 min at room temperature. Finally, after washing with PBS, cells were imaged in five randomly selected fields under a light microscope (Olympus Corporation) at × 100 magnification.

### 5-Ethynyl-2’-deoxyuridine (EdU) incorporation assay

According to the manufacturer’s instructions, EdU Kit (Roche, Mannheim, Germany) was utilized to monitor the proliferation of transfected cells. Zeiss Axiophot Photomicroscope (Carl Zeiss, Oberkochen, Germany) was used to capture representative images.

### Genomic heterogeneity and cancer stemness

Genomic heterogeneity includes the parameters TMB, MATH, tumour ploidy, HRD, LOH, MSI, NEO, and tumour purity [21, 30]. Cancer stemness, including DNAss, EREG-METHss, DMPss, ENHss, RNAss, and EREG.EXPss [31], was calculated using one-class logistic regression algorithms with mRNA expression and methylation signature.

### DNA methylation and alternative splicing

MethSurv (https://biit.cs.ut.ee/methsurv/) was used to evaluate the effects of methylation levels and *PROS1* expression on prognosis in LGG [32], and we used the OncoSplicing website (http://www.oncosplicing.com/) to explore differential alternative splicing events of *PROS1* in LGG [33].

### Survival analysis

The PrognoScan database (http://www.prognoscan.org/) is a large collection of publicly available cancer microarray datasets [34]. The OncoLnc database (www.oncolnc.org), a tool for interactive explorations of survival correlations, contains survival data of 8,647 patients from 21 cancer studies supported by the TCGA. The relationships between *PROS1* expression and patient prognosis (overall survival, disease-specific survival, disease-free interval, and progression-free interval) were visualised with forest plots and Kaplan–Meier curves.

### Analysis of PROS1-interacting genes and proteins

The GeneMANIA database (http://www.genemania.org) was used to construct the *PROS1* interaction network [35]. The STRING database (https://string-db.org/) was used to construct the protein–protein interaction network of PROS1 [36].

### Single-cell analysis

CancerSEA (http://biocc.hrbmu.edu.cn/CancerSEA/home.jsp), a database that aims to comprehensively decode functional states of cancer cells at single-cell resolution, was used to explore *PROS1* functions [37].

### GO, KEGG pathways, and GSEA of *PROS1*

GO and KEGG analyses were applied to explore the biological functions of *PROS1* in LGG. GSEA was used to investigate potential *PROS1* mechanisms. GO, KEGG, and GSEA analyses were analysed using the R package “ClusterProfiler”.

### Tumor immune dysfunction and exclusion (TIDE)

TIDE (http://tide.dfci.harvard.edu/), based on tumour pretreatment expression profiles, can estimate multiple published transcriptomic biomarkers to predict patient responses. It was used to investigate the association between *PROS1* expression and therapy outcomes in clinical studies of immune checkpoint blockade [38].

### Statistical analysis

Most analyses were conducted using R software, and the rest were GraphPad Prism 8.0. Logistic regression, univariate, and multivariate analyses were used to assess the influence of clinical variables on patient survival. Two-tailed P-values less than 0.05 were considered statistically significant.

## Results

### Analysis of *PROS1* expression in patients with LGG

**Fig. 1:**
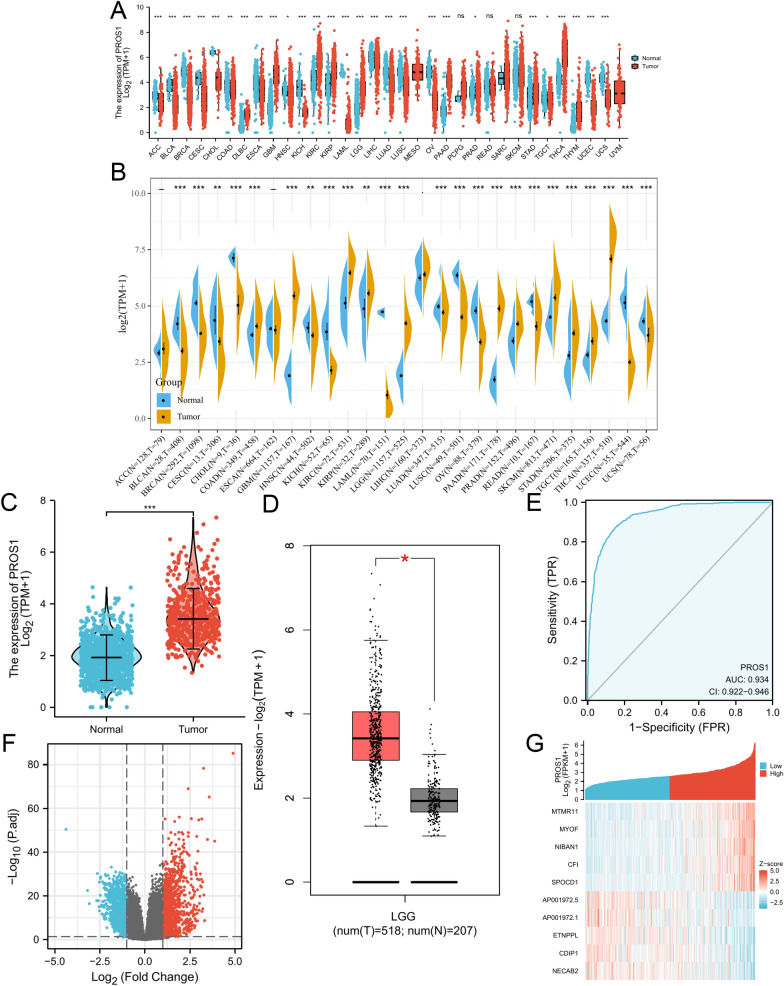
Differential expression levels of PROS1 in different cancers and PROS1-related differentially expressed genes (DEGs). **A**, **B** The expression of PROS1 in multi- types of human cancer based on TCGA cancer and GTEx database, **C**, **D** Differential expression levels of PROS1 in LGG, **E** The ROC curve to test the value of PROS1 to identify LGG tissues was created, **F**, **G** Volcano plots of the DEGs and heat map showing the up-regulated and down-regulated top 5 DEGs

**Fig. 2:**
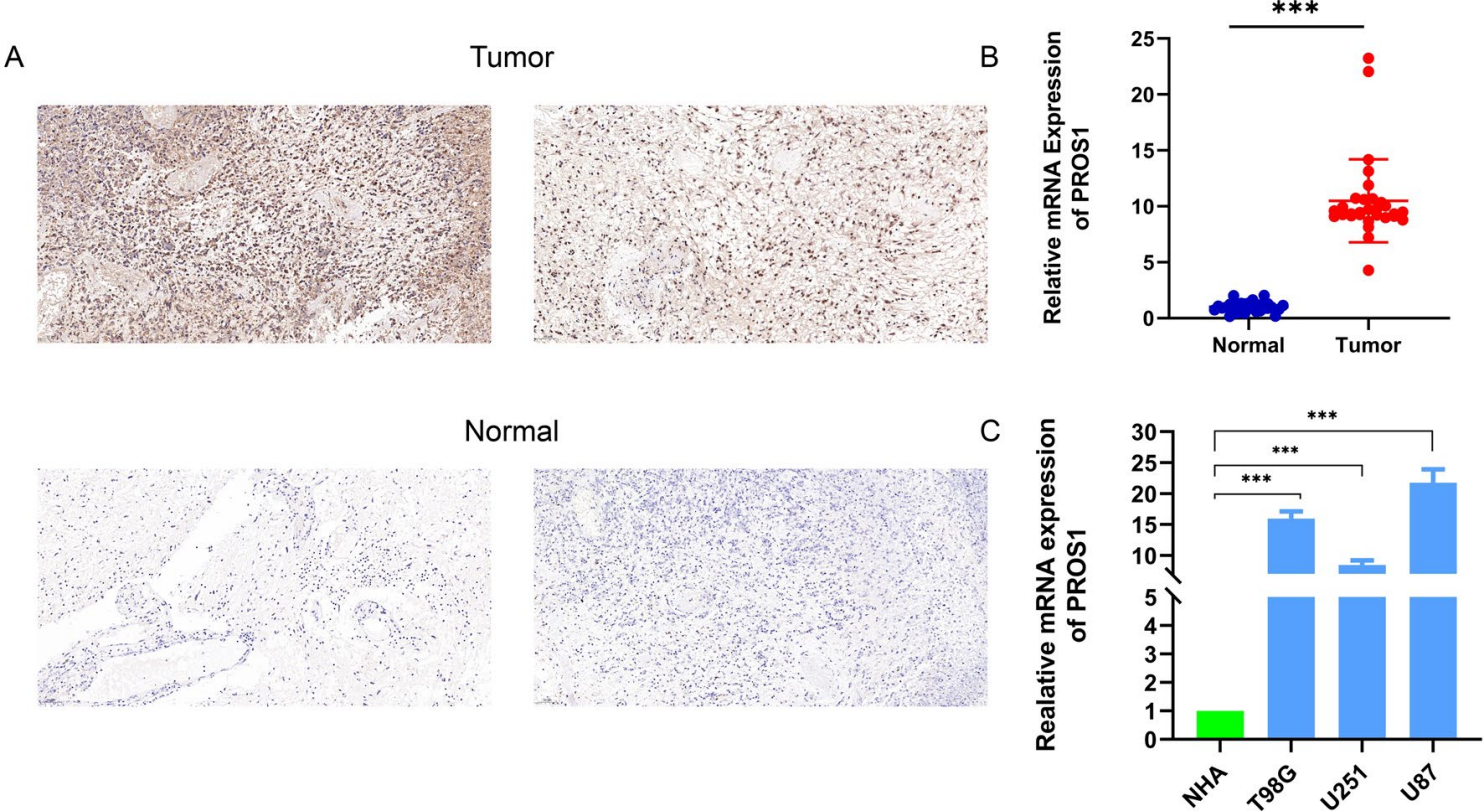
mRNA and protein expression of PROS1 in glioma patients. **A** Immunohistochemical staining of PROS1 was performed in 15 paired tumor samples compared with adjacent normal samples.Scare bars, 50 mM. **B** Realtive mRNA expression of PROS1 in 30 paired tumor samples compared with adjacent normal samples. **C** Realtive mRNA expression of PROS1 in 4 cell lines (NHA, U87, U251 and T98G). ***p < 0.001

To explore possible anti- and pro-carcinogenic roles of PROS1, its mRNA expression was first analysed in human cancer and validated using the Sangerbox tools*.* As shown in Fig. 1A, *PROS1* expression was markedly increased in 11 tumour types (DLBC, GBM, KIRC, KIRP, LGG, PAAD, PRAD, STAD, THCA, THYM, and UCEC) and significantly decreased in 17 tumour types (ACC, BLCA, BRCA, CESC, CHOL, COAD, ESCA, HNSC, KICH, LAML, LIHC, LUAD, LUSC, OV, TGCT, UCEC, and UCS) compared to that in normal samples. However, *PROS1* expression was not significantly different among MESO, PCPG, READ, SKCM, and UVM. Moreover, Sangerbox tools were used to validate mRNA expression results. As shown in Fig. 1B, *PROS1* expression in COAD, GBM, KIRC, KIRP, LGG, PAAD, PRAD, SKCM, STAD, TGCT, and THCA was significantly increased when compared to that in control samples of the genotype-tissue expression (GTEx) project, whereas in BLCA, BRCA, CESC, CHOL, HNSC, KICH, LAML, LUAD, LUSC, OV, READ, UCEC, and USC, *PROS1* expression was substantially decreased. Taken together, *PROS1* was upregulated in GBM, LGG, KIRC, KIRP, PAAD, PRAD, STAD, and THCA, and downregulated in BLCA, BRCA, CESC, CHOL, HNSC, KICH, LAML, LUAD, LUSC, OV, UCEC, and USC, demonstrating that *PROS1* may function as a vital regulator of carcinogenesis in 20 different types of cancer. We additionally analysed *PROS1* expression in 31 types of tissues using the GTEx dataset (Additional file 1: Fig. S1), determined its expression in 21 tumour cell lines using the Cancer Cell Line Encyclopedia database (Additional file 1: Fig. S2), and compared the expression between tumour and normal tissues using The Cancer Genome Atlas (TCGA) database (Additional file 1: Fig. S3). A separate analysis of *PROS1* mRNA levels showed a significant difference between LGG and normal GTEx samples (Fig. 1C). Likewise, in the Gene Expression Profiling Interactive Analysis (GEPIA) database, higher *PROS1* mRNA expression was found in LGG tissues than in normal brain tissues (Fig. 1D). Receiver operating characteristic curves were used to analyse the efficacy of *PROS1* levels to distinguish between LGG samples and normal brain samples. The area under the curve (AUC) of 0.934 (0.922–0.946) suggests that the *PROS1* gene may have the potential to identify LGG tissues. At the protein level, immunohistochemistry stainings were used to investigate the PROS1 expression in 15 paired tumour samples compared with adjacent normal tissues. The analysis demonstrated that PROS1 levels were substantially increased in LGG tissues (Fig. 2A). Moreover, 30 paired samples and 4 cell lines were investigated by qRT-PCR. *PROS1* mRNA expression was significantly upregulated in all 30 tumour samples compared to that in adjacent normal samples (Fig. 2B), as well as in 3 glioma cell lines (T98G, U87, and U251) compared to that in the non-malignant human astrocyte cell line NHA (Fig. 2C).

### Identification of DEGs in LGG

PROS1-high samples and PROS1-low samples from HTSeq-Counts of TCGA-LGG were explored using the R package “DESeq2”. In total, 2,050 DEGs showed statistically significant group differences, including 1,023 upregulated genes and 1,027 downregulated genes (Fig. 1F). The relative expression values of the top five upregulated and downregulated DEGs in these two groups are depicted as a heatmap in Fig. 1G.

### *PROS1* expression in LGG is associated with DNA methyltransferase expression, RNA modification genes, DNA mismatch repair system genes, copy number variations, and the frequency of single nucleotide variations

**Fig. 3:**
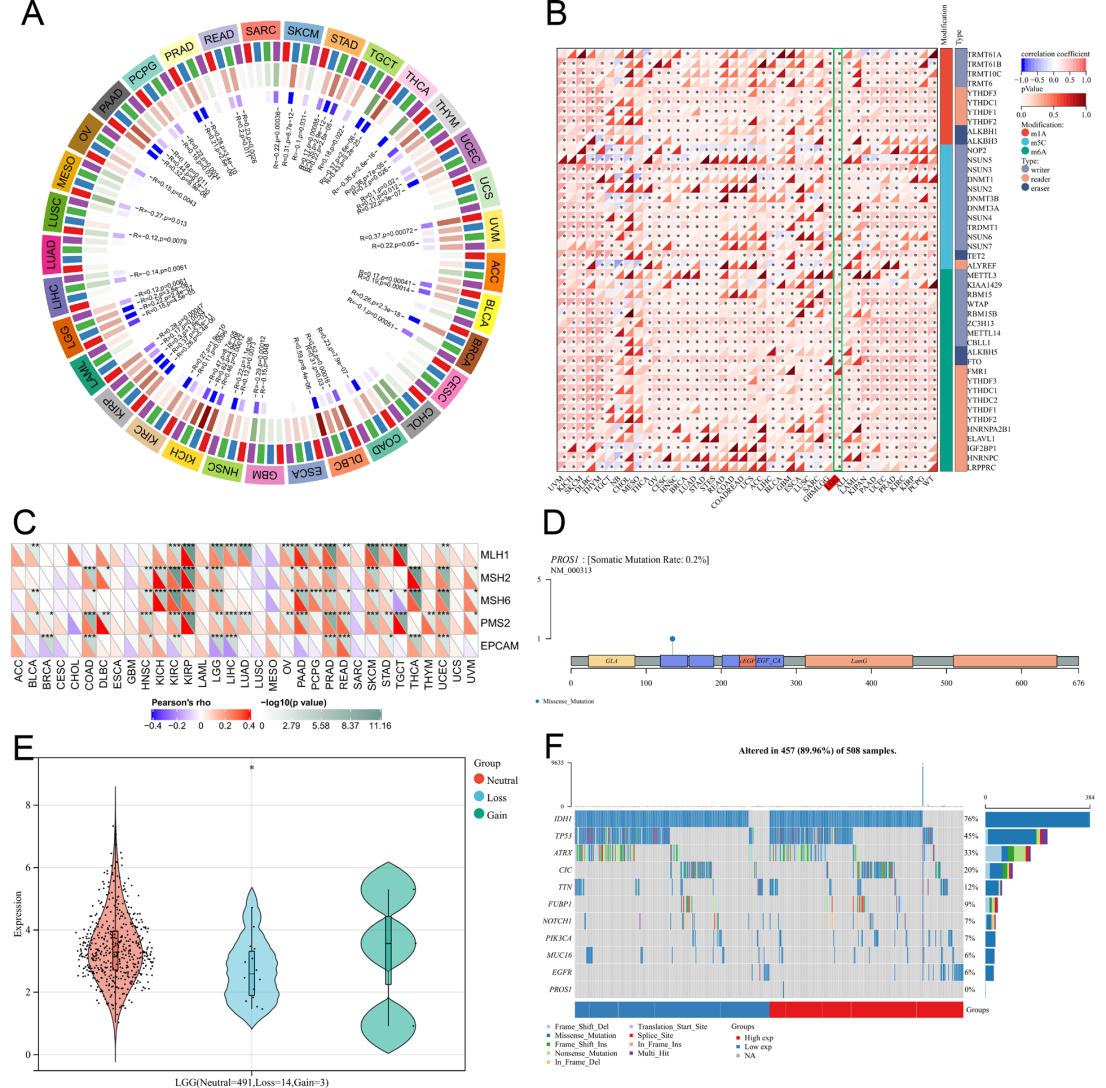
Correlation analysis between the expression of PROS1 and the expression levels of methyltransferases, RNA Modifications Genes, DNA Mismatch Repair System (MMRs) Genes, Copy Number Variation (CNV), and Single nucleotide variation (SNV) frequency. **A** Correlation analysis of PROS1 expression with that of 4 DNA methyltransferases (DNMT1 was colored red, DNMT2 was colored blue, DNMT3a was colored green, and DNMT3b is colored purple) in pan-cancer. **B** Correlation analysis of PROS1 expression with 44 RNA modifications genes (m1A(10)、m5C(13)、m6A(21)) in pan-cancer. **C** Correlation analysis of PROS1 expression with with the mutation levels of 5 MMR genes (MLH1, MSH2, MSH6, PMS2, and EPCAM) in pan-cancer. **D** Lollipop plot displaying mutation distribution and protein domains for PROS1 in cancer with the labeled recurrent hotspots. **E** Correlation analysis of PROS1 expression with CNV alternation in three groups (Neutral = 491, Loss = 14, Gain = 3) tested by by kruskal method. **F** Oncoplot displaying the somatic landscape of low grade gliomas cohort

At the epigenomic level, various epigenetic modifications have the possibility of functional gene modification. DNA methylations such as N1-methyladenosine (m1A), cytosine-5-methylation (m5C), and N6-methyladenosine (m6A) are common epigenetic modifications, so we explored their correlations with *PROS1* expression levels. As shown in Fig. 3A, B *PROS1* expression has a close relationship with the expression of 4 DNA methyltransferase genes (*DNMT1*, *DNMT2*, *DNMT3A*, and *DNMT3B*) and 44 RNA modifications (m1A [n = 10], m5C [n = 13], and m6A [n = 21]) across most human cancers, but especially in patients with LGG, which indicates that *PROS1* may mediate tumorigenesis by epigenetic modification. DNA mismatches are potentially mutagenic and, thus, must be corrected by the mismatch repair system to maintain the integrity of the genetic information. As shown in Fig. 3C, we evaluated the correlation between the mutation levels of five mismatch repair system genes (*MLH1*, *MSH2*, *MSH6*, *PMS2*, and *EPCAM*) and *PROS1*, which suggested that the mismatch repair system may play a critical role in regulating tumorigenesis of *PROS1*. We analysed SNP data to detect variants and their frequencies in LGG. As shown in Fig. 3D, F missense mutations (0.2%) were the main type of SNPs. A frequency analysis of single nucleotide variations comparing groups with high and low *PROS1* expression levels revealed *IDH1*, *TP53*, *ATRX*, *CIC*, *TTN*, *FUBP1*, *MUC16*, *NOTCH1*, *PIK3CA*, and *EGFR* as the top 10 mutated genes, with mutation percentages of 76%, 45%, 33%, 20%, 12%, 9%, 7%, 7%, 6%, and 6%, respectively (Fig. 3F).Moreover, our analysis results showed that *IDH1* and *TP53* were the top2 mutation gene, and mutation frequency of *IDH1* and *TP53* in low PROS1 expression group was more than high PROS1 expression group, which also identified *PROS1* may promote tumour progression and lead to poor prognosis in LGG patients. To identify alterations in copy number variation, data of *PROS1* copy number variation extracted from the TCGA were explored using the R package “copynumber”, and significant sample differences among the three types (491 neutral, 14 loss, and 3 gain) were detected by the Kruskal–Wallis test (Fig. 3E).

### Correlation of *PROS1* expression with DNA methylation and alternative splicing

**Fig. 4:**
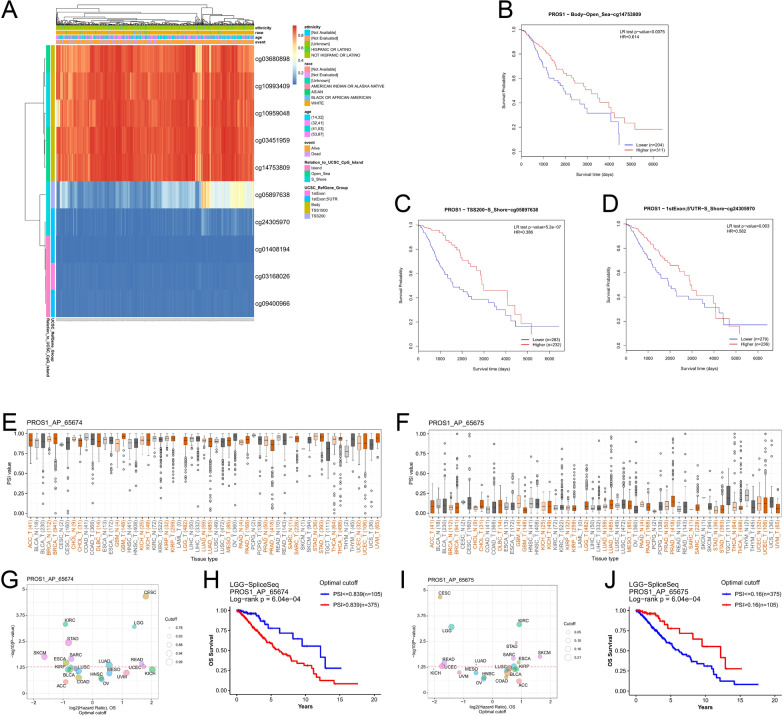
The DNA methylation and Alternative Splicing (AS) of PROS1 in LGG. **A** The visualization between the DNA methylation level and PROS1 expression. **B**–**D** The Kaplan–Meier survival of the 3 promote (cg14753809, cg05897638, and cg24305970) methylation of PROS1. **E**–**J** Two Alternate promoters (PROS1_AP_65674 and PROS1_AP_65675) were found in TCGA SpliceSeq database that related with poor prognosis

As shown above, *PROS1* expression was significantly correlated with the expression of four DNA methyltransferases. To explore the potential mechanism between *PROS1* expression and DNA methylation, MethSurv was utilised to investigate the impact of different methylation levels and *PROS1* expression on the prognosis in LGG. As shown in a heatmap (Fig. 4A), we found high levels of methylation in some of the probe regions (cg03680898, cg10993409, cg10959048, cg03451959, and cg14753809) and low methylation levels in other probe regions (cg05897638, cg24305970, cg01408194, cg03168026, and cg09400966). The survival analysis indicated that cg14753809, cg05897638, and cg24305970 were associated with poor prognosis (Fig. 4B–D). Alternative splicing has been identified as a vital mechanism regulating phenotypic diversity, gene expression, and proteomics; it can be used in biomarker and drug resistance research, as well as a therapeutic target. We used the OncoSplicing website to explore differential alternative splicing events of the *PROS1* gene in LGG. Two alternative promoters (PROS1_AP_65674 and PROS1_AP_65675; Fig. 4E, F) found in the TCGA SpliceSeq database were associated with poor prognosis (Fig. 4G–J).

### *PROS1* expression is associated with genomic heterogeneity and cancer stemness

Genomic heterogeneity and cancer stemness were closely correlated with tumour treatment selection and overall survival. We analysed the association of *PROS1* expression with genomic heterogeneity and cancer stemness in patients with LGG. The results indicated that tumour mutational burden (TMB; R = 0.131, P = 0.00094), mutant-allele tumour heterogeneity (MATH; R =  − 0.117, P = 0.0082), tumour ploidy (R =  − 0.100, P = 0.024), homologous recombination deficiency (HRD; R = 0.140, P = 0.0021), loss of heterozygosity (LOH; R = 0.227, P = 3.31e − 7), DNA methylation-based stemness (DNAss; R = 0.092, P = 0.037), enhancer elements/DNA methylation-based stemness (ENHss; R = 0.097, P = 0.0288), epigenetically regulated DNA methylation-based stemness (EREG-METHss; R = 0.109, P = 0.0138), and RNA expression-based stemness (RNAss; R =  − 0.341, P = 2.933e − 15) were significantly different from those of PROS1 (Additional file 1: Fig. S4). However, there was no significant difference in microsatellite instability (MSI), neoantigen (NEO), tumour purity, differentially methylated probes-based stemness (DMPss), and epigenetically regulated RNA expression-based stemness (EREG.EXPss; Additional file 1: Fig. S5). These data indicate that *PROS1* expression may influence cancer treatment and prognosis by affecting genomic heterogeneity and cancer stemness.

### Correlation between *PROS1* expression and clinical parameters of patients with LGG

To evaluate *PROS1* expression among groups of patients with different clinicopathological characteristics, we analysed 510 LGG samples from the TCGA database with their clinical data. As shown in Additional file 1: Fig. S6 and Table S1, *PROS1* expression was significantly correlated with the WHO grade, *IDH* status, primary therapy outcome, histological type, and outcome measures (overall survival, disease-specific survival, and progression-free interval). All other examined clinical parameters were not significantly correlated with *PROS1* expression (Additional file 1: Fig. S6). The results of the logistic regression analysis indicated that the categorical dependent variable *PROS1* expression was correlated with poor prognostic clinical parameters, including WHO grade, *IDH* status, age, and histological type (Additional file 1: Table S2). Taken together, LGG with high *PROS1* expression may be more likely to progress to a more advanced grade and less susceptible to *IDH1* mutations than LGG with low *PROS1* levels.

### Correlation between *PROS1* expression and the prognosis of patients with LGG

**Fig. 5:**
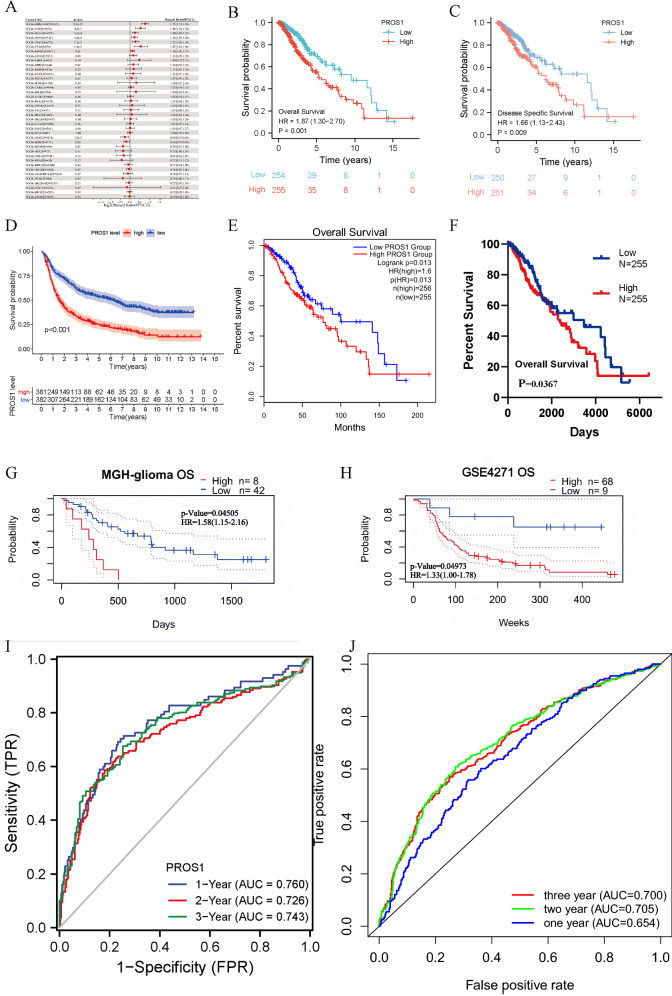
The prognostic value of PROS1 in LGG. **A** Forest plots showing the HRs related to PROS1 expression in pan-cancer. **B**–**H** Kaplan–Meier curves for patients stratified by different expression levels of PROS1 in TCGA (**B**, **C**), CGGA, GEPIA database (**E**), OncoLnc (**F**), PrognoScan database (**G**, **H**). **I**–**J** 1-, 2- and 3 year overall survival ROC curves based on risk score in TCGA (I)and CGGA cohorts (**J**)

**Fig. 6:**
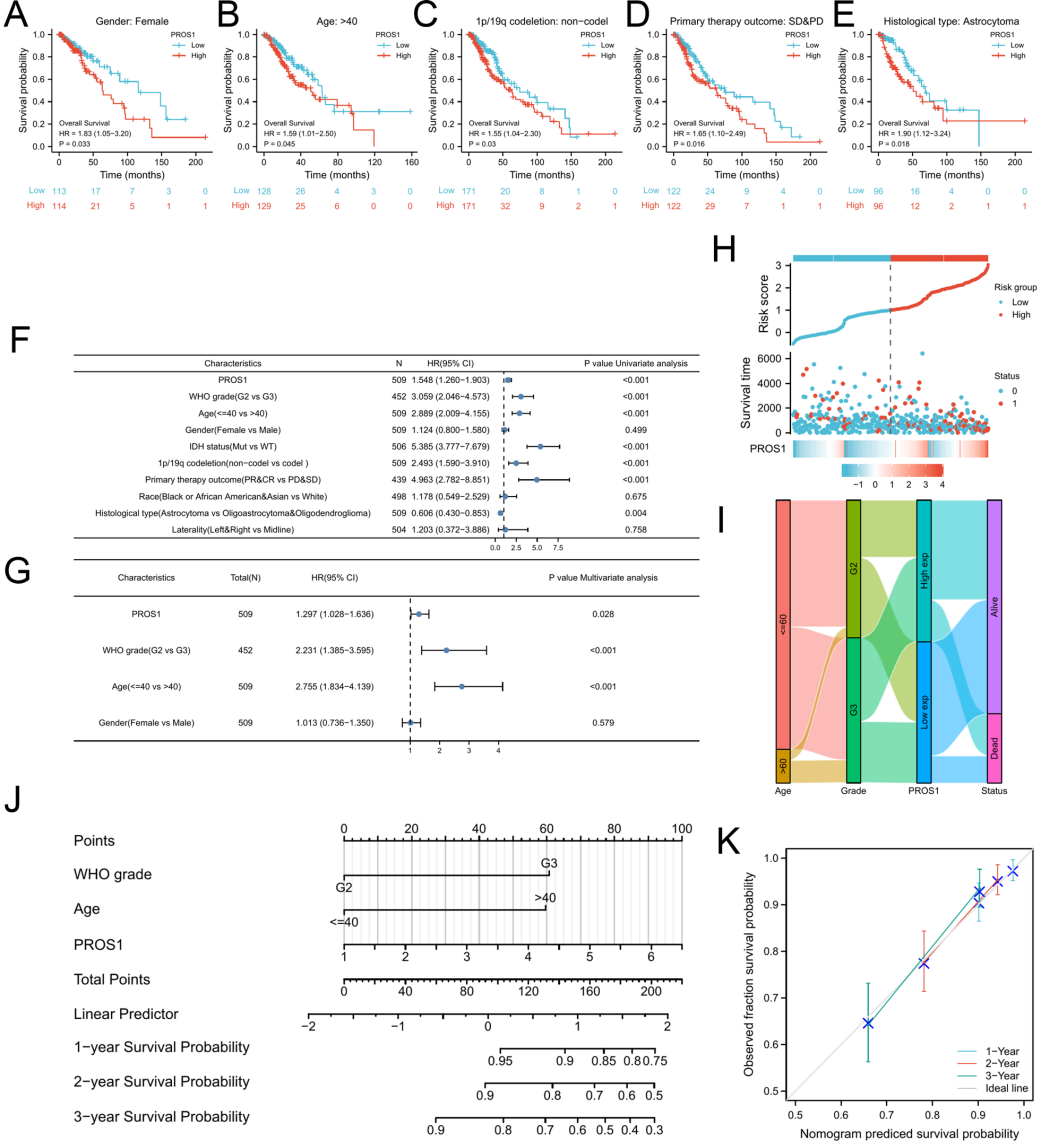
Subgroups prognostic, univariate, multivariate Cox regression,and nomogram analysis of PROS1 in LGG. **A**–**E** Kaplan–Meier curves of high and low PROS1 expression in subgroups, including female (**A**), Age > 40 years (**B**), 1p/19q non-codeletion (**C**), SD&PD (**D**), and astrocytoma (**E**). **F**–**G** The univariate **F** and multivariate Cox regression **G** analysis of PRSO1 in LGG patients. **H**–**I** Risk factor associations diagram **H** and Sankey diagram **I** were used to depicted the overall prognostic trend and living status of the inner relationship. **J** The nomogram for predicting the probability of 1-, 3-, and 5- year OS for LGG patients. **K** Calibration plots of the nomogram for predicting the probability of OS at 1, 3, and 5 years

Since *PROS1* expression levels were closely correlated with LGG tumour progression, we subsequently explored their prognostic significance. As shown in the forest plot (Fig. 5A), we first analysed the correlation between *PROS1* expression and overall survival in 33 tumours from the TCGA database. The results indicated that high *PROS1* expression was related to poor survival in several cancers (LGG, BCLA, STES, STAD, and UVM), and the pan-cancer analysis demonstrated similar results for disease-specific survival, disease-free interval, and progression-free interval (Additional file 1: Figs. S7–S9). Next, we analysed the correlations of *PROS1* expression with overall survival and disease-specific survival (Fig. 5B, C respectively). These results were validated by data from various databases and websites, including the Chinese Glioma Genome Atlas (CGGA; Fig. 5D), GEPIA database (Fig. 5E), OncoLnc database (Fig. 5F), and PrognoScan database (Fig. 5G, H). Based on receiver operating characteristic curves of TCGA (Fig. 5I) and CGGA (Fig. 5G) data, the associations of *PROS1* expression with 1-, 2-, and 3 year were explored. The results suggested that *PROS1* expression had a moderate prognostic value. Next, we conducted subgroup analyses of overall survival based on clinicopathological characteristics. The prognosis of LGG patients with high *PROS1* levels was poor in women, age > 40 years, 1p/19q non-codeletion, SD&PD, and astrocytoma subgroups (Fig. 6A–E). Univariate (Fig. 6F and Additional file 1: Table S3) and multivariate (Fig. 6G and Additional file 1: Table S3) Cox regression analyses showed that *PROS1* expression, WHO grade, and age were independent prognostic factors for overall survival of patients with LGG. Risk factor association (Fig. 6H) and Sankey (Fig. 6I) diagrams were used to visualise the overall prognostic trends of the groups with high and low *PROS1* expression, as well as the relationships among the three independent factors in living status. Finally, a nomogram was constructed based on *PROS1* expression, age, and WHO grade as a quantitative tool for predicting the prognosis of patients with LGG (Fig. 6J). The prediction efficiency of the nomogram was evaluated using the C-index (0.754, confidence interval: 0.727–0.782), which implied that this model had moderate prediction accuracy. Consistent with the former results, the agreement between predictions and observations was good (Fig. 6K).

### Identification of PROS1-interacting genes and proteins

**Fig. 7:**
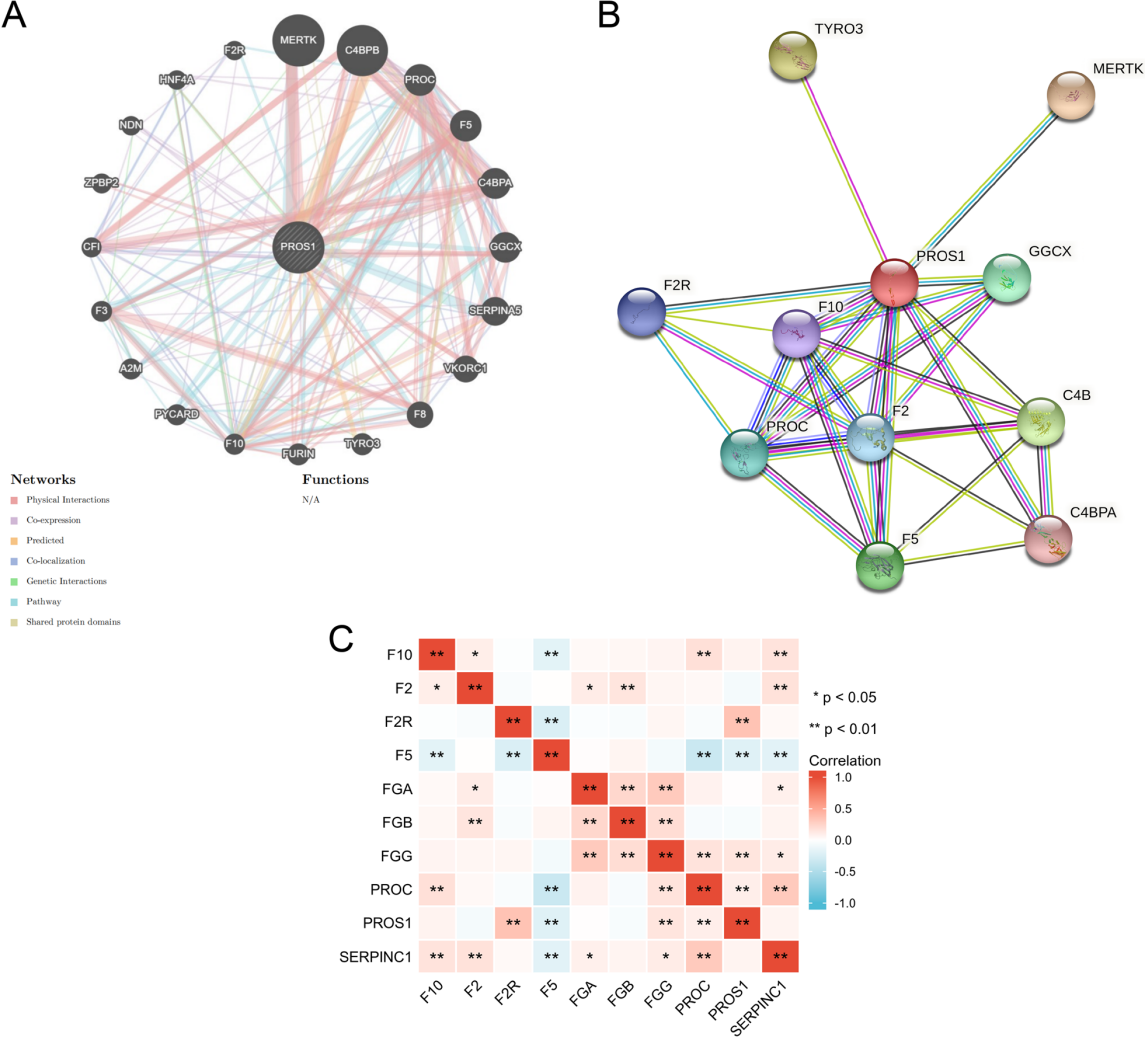
Identification of PROS1-Interacting Genes and Proteins. **A** The gene–gene interaction network of PROS1 was constructed using GeneMania. **B** The PPI network of PROS1 was generated using STRING. **C** A heat map shows the correlations between PROS1 and icoagulation related genes in LGG

The GeneMANIA database was used to analyse the gene–gene interaction network of *PROS1* and identify altered neighbouring genes. The results showed that 20 genes were most closely related to *PROS1*, including *MERTK*, *C4BPB*, *F5*, and *F8* (Fig. 7A). Similarly, the STRING database was used to assess the PROS1 protein–protein interaction network which contained 29 edges and 11 nodes, including TYRO3, PROC, and GGCX (Fig. 7B). Taking the above results into account, the relationships between coagulation-related genes and *PROS1* was explored in LGG data from the TCGA. The significant results of this analysis demonstrated that in LGG, *PROS1* was positively correlated with *F2R*, *FGG*, and *PROC* but negatively correlated with *F5* (Fig. 7C).

### Analysis of long noncoding RNA (lncRNA)- transcription factor (TF)-*PROS1* triplets

**Fig. 8:**
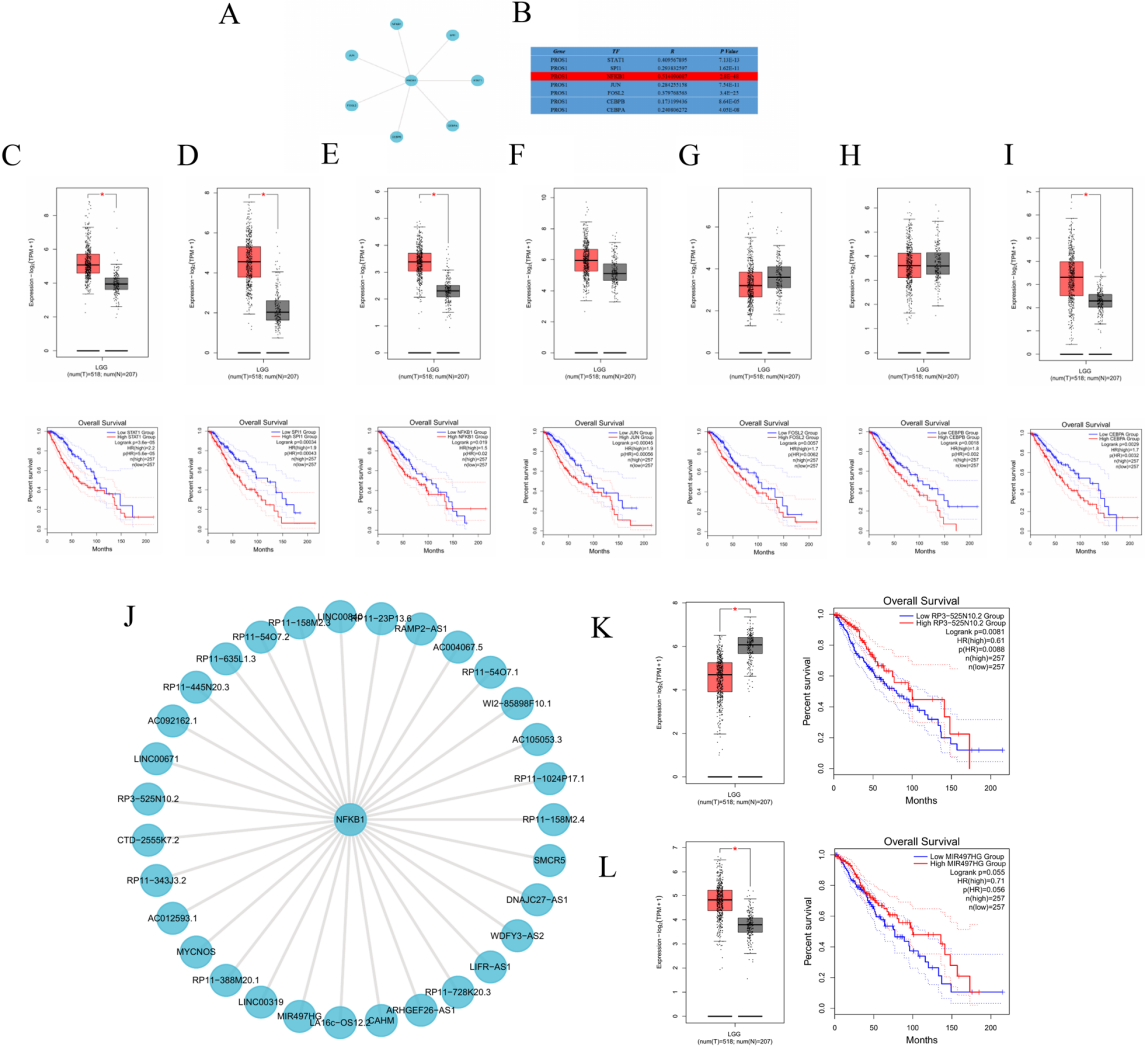
Identification LncRNA-TF-PROS1 Triplet in LGG. **A** The TF-PROS1 regulatory network. **B** The expression correlation between predicted TFs and PROS1 in LGG. **C**–**I** The expression and prognosis analysis of TFs in LGG Using GEPIA database. **J** The LncRNA-NFKB1 regulatory network. **K**–**L** The expression and prognosis analysis of possible LncRNAs in LGG Using GEPIA database

To better understand the potential regulatory mechanisms of *PROS1* in LGG, we examined the regulation of *PROS1* expression by lncRNA-TF-gene triplets. We used the LncMAP database to explore possible *PROS1-*containing triplet formations in LGG. The results showed that seven TFs may modulate *PROS1* expression in LGG (Fig. 8A). To identify the TFs most likely regulating the *PROS1* gene, we conducted an expression, prognosis, and correlation analysis of these seven TFs (Fig. 8B). Among the four TFs (*STAT1*, *SPI1*, *NFKB1*, and *CEBPA*) with high expression and prognostic significance in LGG (Fig. 8C–I), the correlation coefficient was the highest (R = 0.514) between *NFKB1* and *PROS1*. Subsequently, we explored lncRNAs possibly acting on *NFKB1* and identified 31 lncRNAs that can interact with *NFKB1* (Fig. 8J). Differential expression and prognostic analyses were used to determine the target lncRNAs. Ultimately, the two lncRNAs RP3-525N10.2 and MIR497HG were significantly differentially expressed in LGG (Fig. 8K–L), but only RP3-525N10.2 was significantly downregulated, suggesting a good prognosis. In summary, we identified the triplet RP3-525N10.2-*NFKB1*-*PROS1* and, considering the downregulated expression of RP3-525N10.2 in LGG, suggest its possible role as a tumour suppressor gene affecting the prognosis in LGG. We propose that the lncRNA RP3-525N10.2 may act as a “decoy” that binds to the TF *NFKB1* or guide the TF, and prevents its association with the target *PROS1*, which can reduce *PROS1* expression, thereby improving the prognosis in patients with LGG.

### Gene ontology (GO), kyoto encyclopedia of genes and genomes (KEGG) pathways, and gene set enrichment analysis (GSEA) of *PROS1* in patients with LGG

**Fig. 9:**
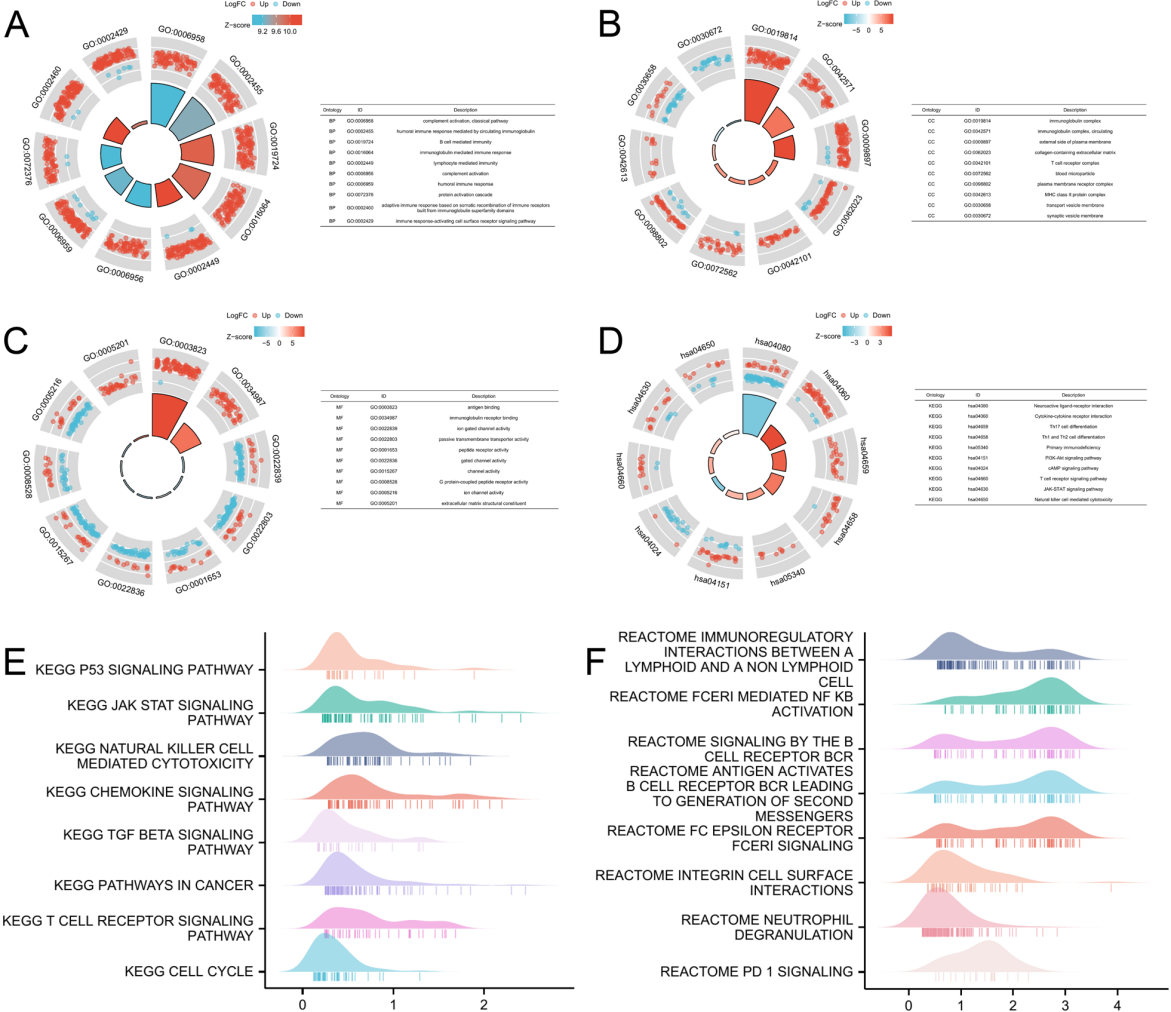
Significantly enriched GO, KEGG and GSEA annotations of PROS1 related genes in LGG. **A**–**D** The top 10 significant terms of BP (**A**), MF (**B**), CC **C** and KEGG **D** enrichment analysis were depicted by circular plot. **E**, **F** The top 8 pathways of GSEA enrichment analysis were depicted by ridge plot, including KEGG **E** and Reactome pathways (**F**)

GO and KEGG enrichment analyses were used to investigate *PROS1*-related biological pathways and functions. Circular plots depict the top 10 significant terms of BP, MF, CC, and KEGG enrichment analyses (Fig. 9A–D). Notably, in all analysis results, *PROS1* was enriched in immune response-related processes or pathways, such as complement activation, B cell-mediated immunity, immunoglobulin-mediated immune response, and lymphocyte-mediated immunity in BP term, immunoglobulin complex, T-cell receptor complex, and MHC class II protein complex in CC term, and immunoglobulin receptor binding in MF term. In the KEGG pathway analysis, several immune-related pathways were highly correlated with *PROS1* expression, including Th17 cell differentiation, Th1 and Th2 cell differentiation, primary immunodeficiency, T-cell receptor signalling pathway, and natural killer (NK) cell-mediated cytotoxicity. Consistent with the results of the GO and KEGG analyses, GSEA, including KEGG (Fig. 9E) and Reactome (Fig. 9F) pathways, showed that the top eight signalling pathways affected by *PROS1* were enriched mainly in immune-related activities, including NK cell-mediated cytotoxicity, T-cell receptor signalling pathways, immunoregulatory interactions between lymphoid and non-lymphoid cells, signalling by the B-cell receptor BCR, and programmed death 1 (PD1) signalling. These results strongly imply that *PROS1* participates in the regulation of immune responses in LGG.

### Single-cell analysis of *PROS1* functions in patients and experimental verification in GBM cell lines

**Fig. 10:**
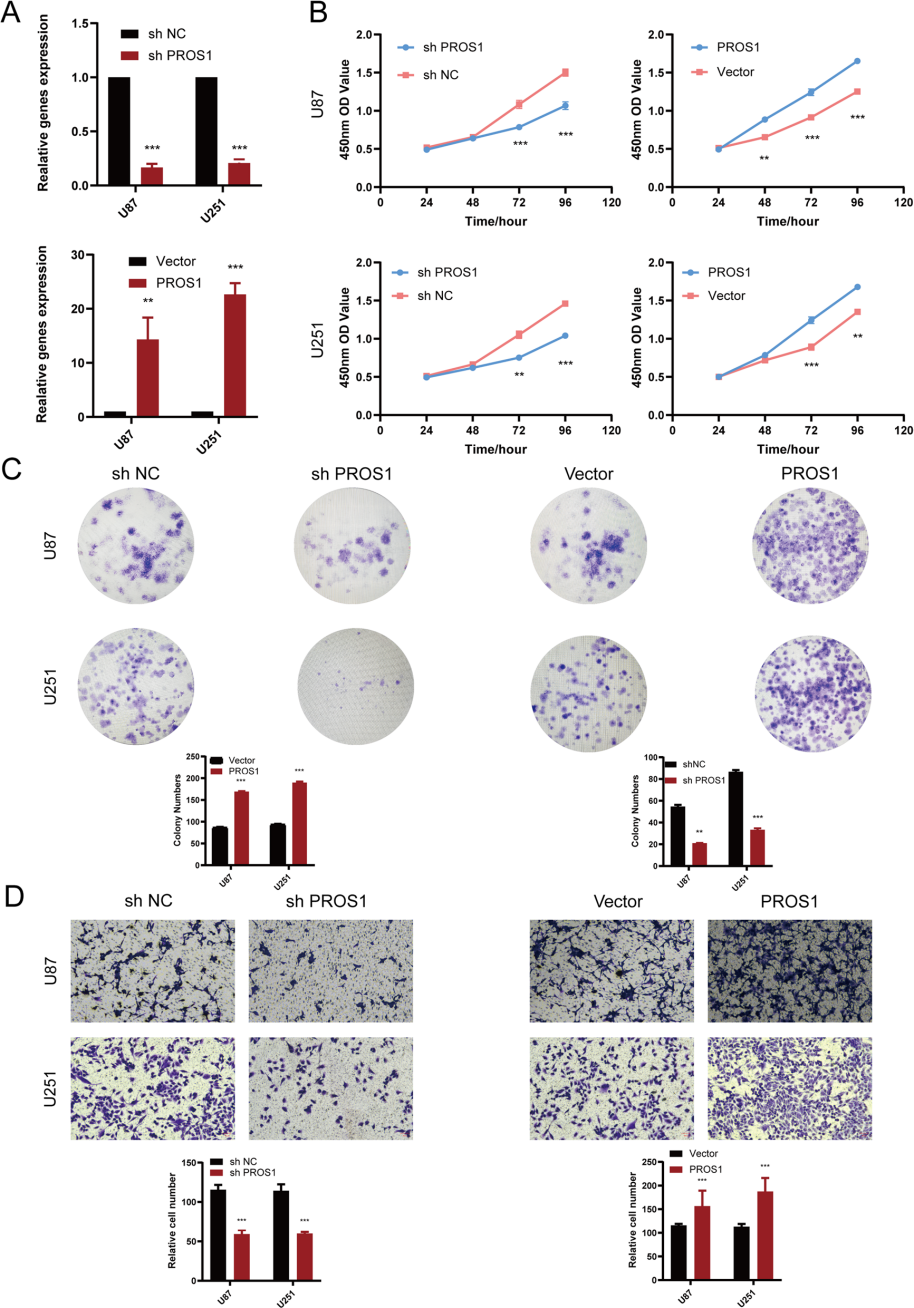
PROS1 increased the proliferation, invasion of GBM cells in vitro. **A** qRT-PCR assays were applied to analyse the expression level of PROS1 after transfection by sh PROS1 or PROS1 overexpression vector for 24 h in U87 or U251 cells. **B** Cell viability of U87 or U251 cells after knocking down or overexpressing PROS1 was determined using CCK8 assays. **C** Colony formation assay were performed in transfected U87 or U251 cells to evaluate cell proliferation ability. **D** Transwell assays were performed in transfected U87 or U251 cells to evaluate cell invasion ability (Magnification: × 100). *P < 0.05, **P < 0.01, ***P < 0.001, ****P < 0.0001. Error bars indicate mean ± SD

**Fig. 11:**
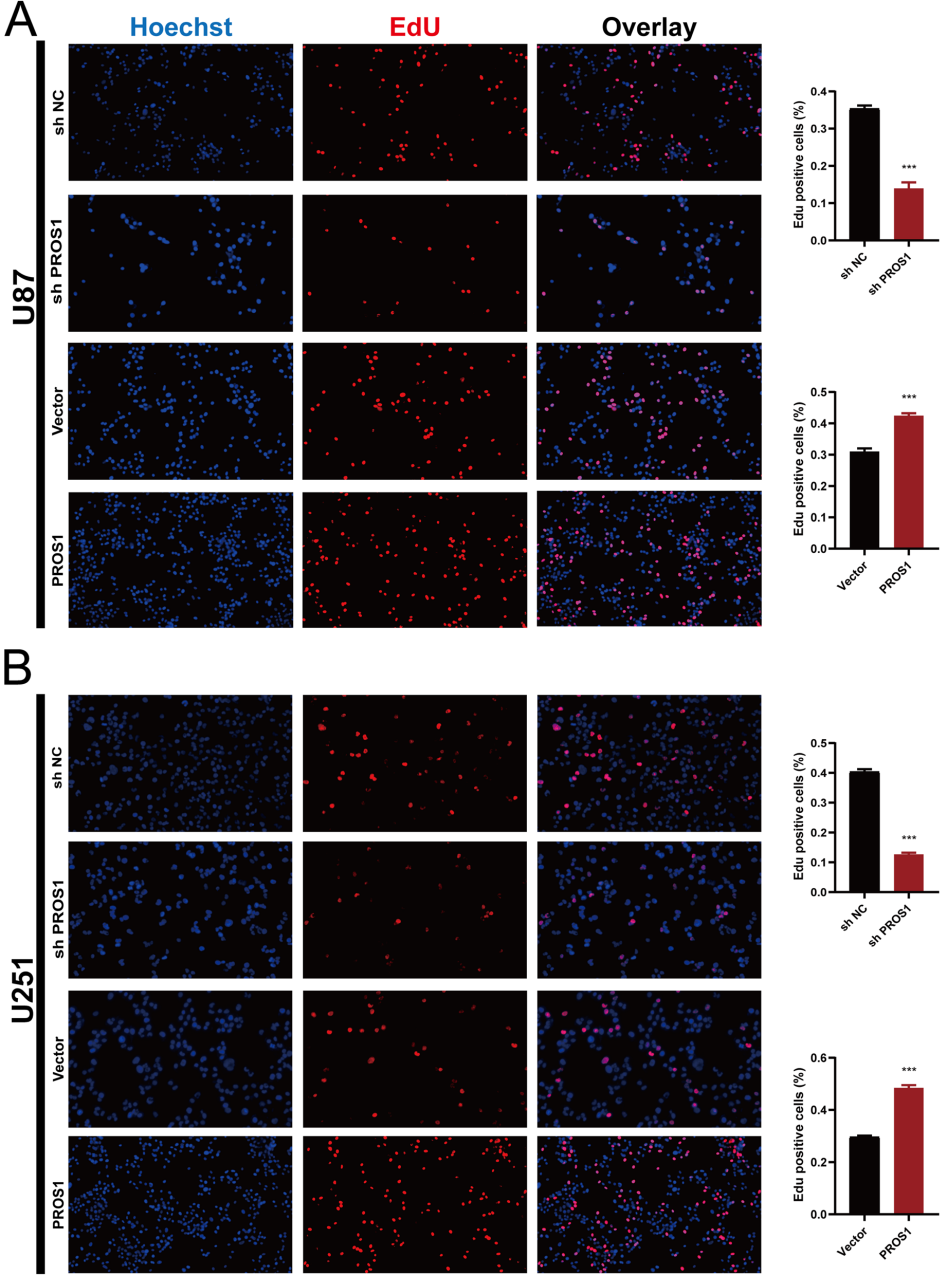
5-Ethynyl-2’-deoxyuridine (EdU) staining to detect cell proliferation. **A** U87 cells were treated with EdU for 6 h prior to click reaction. **B** U251 cells were treated with EdU for 6 h prior to click reaction. Data analysis was performed to calculate the signal intensity in EdU-positive cells based on individual DAPI signal and displayed in the right graph. *P < 0.05, **P < 0.01, ***P < 0.001, ****P < 0.0001.Error bars indicate mean ± SD

To explore the functions of *PROS1* in LGG tissues at the single-cell level, CancerSEA was used to explore phenotypes possibly regulated by *PROS1*. As shown in t-SNE and box plots (Additional file 1: Fig. S10), *PROS1* was upregulated at the single-cell level in EXP0059 glioma cell groups. The results of the functional analyses (Additional file 1: Fig. S10) demonstrated that *PROS1* positively influenced cell invasion in two glioma single-cell sequencing datasets (BCH1126 and BCH836). As shown in Fig. 10, CCK8 assays depicted that knocking down PROS1 expression significantly reduced the proliferation ability of U87 cells and U251 cells; conversely, overexpressing PROS1 increased cell proliferation (Fig. 10B). Transwell assays were used to assess the invasion ability of cells. PROS1 knockdown resulted in a lower U87 cell and U251 cell invasion rate, while PROS1 over-expression showed the opposite results (Fig. 2D). In addition, colony formation assay (Fig. 10C) and 5-Ethynyl-2’-deoxyuridine (EdU) incorporation assay (Fig. 11) also suggested that knockdown of PROS1 inhibited cell proliferation. In contrast, over-expression of PROS1 increased cell proliferation rate. In summary, PROS1 increased the proliferation and invasion of GBM cells.

### *PROS1* is correlated with immune cell infiltration and tumour microenvironment in LGG

**Fig. 12:**
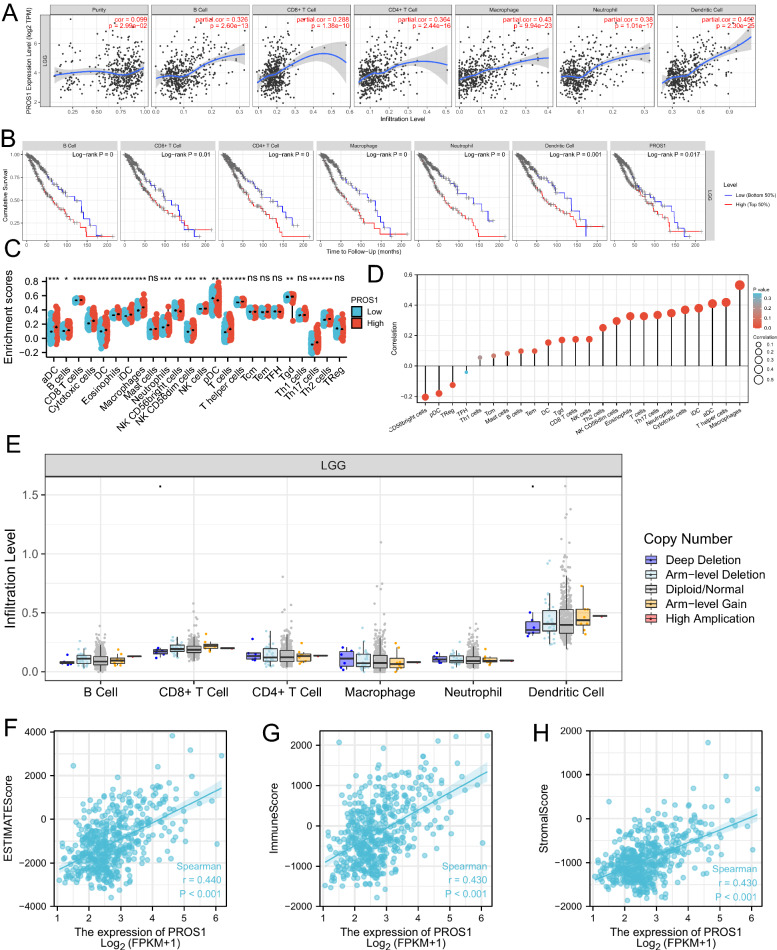
The relationship of immune cell infiltration and tumor micro-environment with PROS1 level in LGG. **A**, **B** The correlation between 6 immune related cells and PROS1 expression **A** and prognosis (B) in LGG patients. **C**, **D** The relationship between PROS1 expression and the immune infiltration analyzed by ssGSEA and depicted by Lollipop Chart and correlation diagrams. **E** The infiltration level of various immune cells under different copy numbers of PROS1 in LGG. **F**–**H** The correlation of PROS1 expression level with immune score, stromal score, as well as ESTIMATE score

The functional enrichment results indicated that *PROS1* may play a critical role in the immune system. Therefore, we investigated the relationships between *PROS1* expression and immune cell infiltration. According to analyses of the Tumor Immune Estimation Resource (TIMER) database, six types of immune cells (B cells, CD8 + T cells, CD4 + T cells, macrophages, neutrophils, and dendritic cells) were associated with *PROS1* expression and prognosis in patients with LGG (Fig. 12A, B). Further analyses using the ssGSEA (Fig. 12C, D) showed that *PROS1* expression levels were significantly positively associated with the infiltration of B cells, Tem, DC, Tgd, CD8 + T cells, NK cells, Th2 cells, NK CD56dim cells, eosinophils, T cells, Th17 cells, neutrophils, cytotoxic cells, iDC, aDC, T helper cells, and macrophages, but significantly negatively correlated with infiltration of CD56bright cells, pDC, and TReg. Moreover, various copy numbers of *PROS1* were not significantly correlated with immune cell infiltration levels in LGG (Fig. 12E). To analyse the possible influence of *PROS1* expression on the tumour microenvironment during tumour development, we analysed immune and stromal scores of LGG samples using the R package “Estimate”. As shown in Fig. 10F, H *PROS1* was positively correlated with immune score (R = 0.440, P < 0.001), stromal score (R = 0.430, P < 0.001), and estimated immune score (R = 0.440, P < 0.001).

### Correlation of *PROS1* expression with the expression of immune cell markers, immune checkpoint inhibitors, and immunomodulators

To better understand the interaction between *PROS1* expression and immune responses, the TIMER and GEPIA databases were utilised to validate the association between *PROS1* expression and immune cell markers in LGG. The genes listed in Table 1 represent various immune cells, including B cells, T cells, CD8 + T cells, monocytes, tumour-associated macrophages (TAMs), M1 macrophages, M2 macrophages, neutrophils, NK cells, and dendritic cells. In LGG, expression of *PROS1* was significantly correlated with that of most immune cell markers (Table 1). Some studies have shown that *PROS1* can influence the activation of TAMs [8, 17–20]; therefore, we explored the relationships between *PROS1* expression and TAMs, including microglia and monocyte-derived macrophages (Table 1). According to the analyses of the TIMER and GEPIA databases, *PROS1* expression levels had a significant positive correlation with 10 of the 13 immune cell markers in LGG (Additional file 1: Fig. S11).

**Table 1:** Correlation analysis between *PROS1* and gene markers of immune cells in TIMER and GEPIA

Immune cell	Biomarker	TIMER		GEPIA	
		R value	p value	R value	p value
B cell	CD19	0.20***	7.11E − 06	0.24***	4.2E − 08
	CD79A	0.11*	1.46E − 02	0.16***	2.3E − 04
T cell	CD3D	0.38***	1.01E − 18	0.34***	1E − 15
	CD3E	0.39***	2.59E − 20	0.41***	6.4E − 22
	CD2	0.44***	1.93E − 26	0.47***	2.6E − 30
CD8 + T cell	CD8A	0.19***	9.38E − 06	0.22***	2.9E − 07
	CD8B	0.18***	2.95E − 05	0.21***	1.9E − 06
CD4 + T cell	CD4	0.41***	6.8E − 12	0.41***	6.8E − 22
Monocyte	CD86	0.33***	8.38E − 15	0.36***	3.1E − 17
	CSF1R	0.21***	1.55E − 06	0.23***	1.2E − 07
TAM (general)	CCL2	0.35***	6.00E − 16	0.35***	4.5E − 16
	CD68	0.42***	3.5E − 25	0.43***	3.5E − 25
	IL10	0.34***	4.17E − 15	0.37***	1.1E − 18
M1	IRF5	0.30***	5.12E − 12	0.31***	3.7E − 13
	PTGS2	0.04*	3.87E − 02	0.091**	3.9E − 02
	NOS2	− 0.13**	2.3E − 03	− 0.11**	1.4E − 02
M2	CD163	0.45***	4.3E − 24	0.42***	4.3E − 24
	VSIG4	0.30***	8.18E − 12	0.31***	2.5E − 13
	MS4A4A	0.44***	2.8E − 29	0.47***	2.8E − 29
Neutrophils	CEACAM8	− 0.01	8.2E − 01	0.0019	9.7E − 01
	ITGAM	0.28***	8.97E − 11	0.29***	8.6E − 12
	CCR7	0.33***	3.68E − 14	0.34***	6.8E − 16
Natural killer cell	KIR2DL1	0.12**	5.1E − 03	0.16***	2.7E − 04
	KIR2DL3	0.22***	3.79E − 07	0.25***	1.2E − 08
	KIR2DL4	0.23***	8.55E − 08	0.28***	1.8E − 10
	KIR3DL1	0.11*	1.3E − 02	0.16***	3E − 04
	KIR3DL2	0.10*	1.9E − 02	0.17***	1.3E − 04
	KIR3DL3	0.01	9.7E − 01	0.019	6.6E − 01
	KIR2DS4	0.18***	3.52E − 05	0.19***	1.1E − 05
Dendritic cell	HLA-DPB1	0.49***	1.6E − 33	0.5***	1.6E − 33
	HLA-DQB1	0.42***	6.5E − 15	0.33***	6.5E − 15
	HLA-DRA	0.52***	4.6E − 38	0.53***	4.6E − 38
	HLA-DPA1	0.52***	7.7E − 38	0.52***	7.7E − 38
	CD1C	0.36***	8.18E − 17	0.34***	8.2E − 16
	NRP1	0.49***	3.43E − 33	0.5***	1.5E − 34
	ITGAX	0.23***	1.51E − 07	0.23***	1.2E − 07
Microglia	CX3CR1	0.216***	7.78E − 07	0.23***	1.6E − 07
	P2RY12	0.075	8,75E − 02	0.1	0.02
	ITGAX	0.229***	1.51E − 07	0.23***	1.2E − 07
	FCGR1A	0.29***	2.34E − 11	0.26***	3.9E − 09
	TMEM119	0.205***	2.6E − 06	0.23***	6.3E − 08
	CD33	0.331***	1.25E − 14	0.32***	1E − 13
Monocyte-derived Macrophages	MRC1	− 0.099	2.4E − 02	− 0.061	0.16
	CD163	0.45***	P < 0.001	0.42***	4.3E − 24
	CD14	0.362***	1.23E − 17	0.38***	7.2E − 19
	TEK	− 0.033	4.49E − 01	0.0087	0.84
	THBD	0.249***	1.14E − 08	0.29***	3.7E − 11
	ICAM1	0.429***	1.69E − 24	0.44***	2.2E − 26
	ITGA4	0.47***	P < 0.001	0.49***	1.4E − 32

*p < 0.05, **p < 0.01, ***p < 0.001

**Fig. 13:**
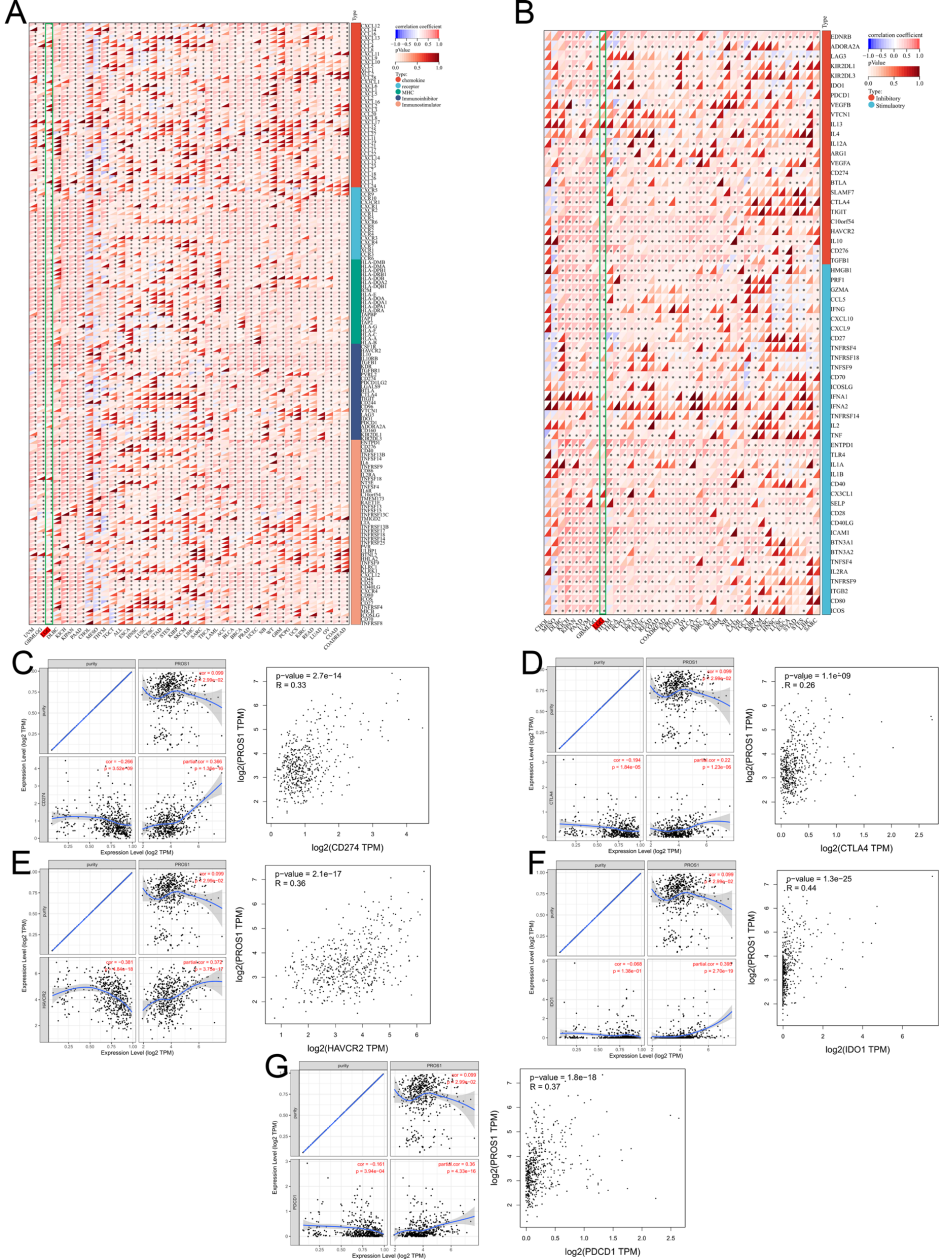
Correlation Between PROS1 expression and Immune checkpoint inhibitors and immunomodulators. **A** Correlation between PROS1 and 150 immunomodulators ((chemokine (41), receptor (18), MHC (21), Immunoinhibitor (24) and Immunostimulator (46)). **B** Correlation analysis between PROS1 expression in Pan-cancer and immune checkpoint gene expression. **C**–**G** Correlation of SEMA3F expression with CD274 (**C**), CTLA4 (**D**), HAVCR (**E**), IDO1 (**F**), and PDCD1 **G** expression in LGG. *Significant correlation P < 0.05, **Significant correlation P < 0.01, ***Significant correlation P < 0.001

The correlation analysis between immunomodulators and *PROS1* expression may reveal the types of cancers that benefit from immunotherapy targeting *PROS1*. In LGG, *PROS1* was positively correlated with most of the 150 analysed immunomodulators (Fig. 13A). Based on the improved understanding of the functions of novel human immune checkpoint inhibitors [21], 60 immune checkpoint genes (24 inhibitory, 36 stimulatory) were explored regarding their correlations with *PROS1* in different cancer types (Fig. 13B). Notably, more than 50 immune checkpoint markers were significantly associated with *PROS1* expression in LGG. The scatter plots of five selected common immune checkpoint genes (*CD274*, *CTLA4*, *HAVCR2*, *IDO1*, and *PDCD1*) in the TIMER and GEPIA databases are shown in Fig. 11C–G. Collectively, these results strongly imply that *PROS1* may play a critical role in LGG immunity and may be a potential immunotherapy target.

### Association between *PROS1* expression and therapy outcomes in clinical studies of immune checkpoint blockade (ICB)

**Fig. 14:**
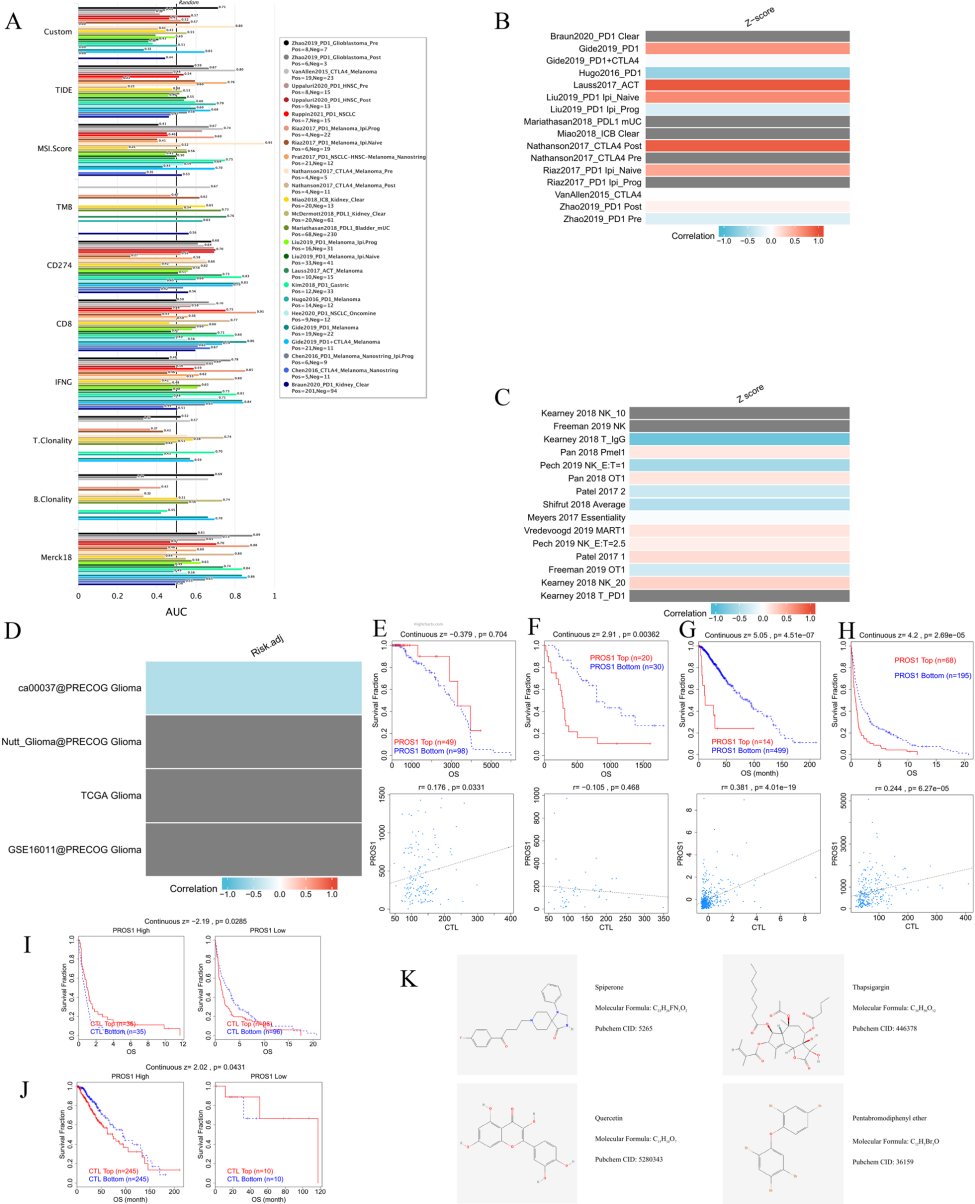
Correlation between PROS1 expression and therapy outcome in clinical studies of immune checkpoint blockade. **A** Bar plot showing the biomarker relevance of PROS1 compared to standardized cancer immune evasion biomarkers in immune checkpoint blockade (ICB) sub-cohorts. The area under the receiver operating characteristic curve (AUC) was applied to evaluate the predictive performances of the test biomarkers on the ICB response status. **B**, **C** Heatmap of PROS1 associations with lymphocyte-mediated tumor killing in CRISPR screens **C** and outcomes in ICB sub-cohorts (**B**). **D**–**H** 4 glioma cohorts (ca00037@PRECOG, Nutt_Glioma@PRECOG, TCGA, and GSE16011@PRECOG) expression levels of MXD3 were correlated with OS and the level of CTL. **I**–**J** CTL expression levels of GSE16011@PRECOG cohort was related with overall survival in PROS1 mRNA expression **I** and copy number alteration data (**J**). **K** 4 drugs were the peak drug candidates based on DSigDB database drug molecules

Based on the predictive power of response outcomes and overall survival among ICB subcohorts, we evaluated the relevance of *PROS1* as a biomarker by comparing its performance with those of standardised biomarkers. We found that *PROS1* alone had an AUC value > 0.5 in 8 of the 20 examined ICB subcohorts (Fig. 14A). *PROS1* exhibited a higher predictive value than B.Clonality, which gave AUC values > 0.5 in 6 ICB subcohorts. However, as a predictive biomarker, *PROS1* was comparable to the combination of TMB score and B.Clonality (AUC > 0.5 in 8 ICB subcohorts) but inferior to MSI, CD27A, TIDE, IFNG, Merck18, and CD8. Our results also indicated that high *PROS1* expression was correlated with worse PD1 outcomes in kidney renal clear cell carcinoma (Braun2020_PD1 Clear), melanoma (Liu2019_PD1 Ipi_Naive, Gide2019_PD1, and Riaz2017_PD1 Ipi_Naive), PD-ligand 1 (LI) (BladderMariathasan2018_PDL1 mUC), ACT melanoma (Lauss2017_ACT), and CTLA4 (Nathanson2017_CTLA4 Post) but achieved good CTLA4 therapeutic outcomes in melanoma (Nathanson2017_CTLA4 Pre), and kidney renal clear cell carcinoma (Miao2018_ICB Clear) cancer cohorts (Fig. 14B). Screening results of knockout phenotypes also implied that *PROS1* knockout may positively impact lymphocyte-mediated tumour killing in MC38 colon cancer (Kearney2018_T_PD1) models (Fig. 14C). Moreover, the MXD3 expression levels were correlated with overall survival (Fig. 14D–H) in 3/4 of the glioma cohorts (Nutt_Glioma@PRECOG, TCGA, and GSE16011@PRECOG) and likewise with CTL levels (Fig. 14D–H) in 3/4 of the glioma cohorts (ca00037@PRECOG, TCGA, and GSE16011@PRECOG). CTL expression levels in the GSE16011@PRECOG cohort were also correlated with overall survival in *PROS1* mRNA expression and copy number alteration data (Fig. 14I, J). Finally, the Enrichr platform was used to identify drug molecules, collected from the DSigDB database, targeting PROS1. The results showed that spiperone PC3, thapsigargin PC3, quercetin CTD 00006679, and pentabromodiphenyl ether CTD 00003077 are the four drug molecules with which most DEGs interact (Fig. 14K).

## Discussion

In patients with LGG, the correlations of *PROS1* expression with prognosis and multi-omics data have not been described yet. This is the first comprehensive evaluation of *PROS1*-related mechanisms possibly underlying carcinogenesis in patients with LGG. In this study, we first analysed *PROS1* gene expression across various human cancers with a focus on LGG using in vitro techniques (qRT-PCRs with 30 pairs of tumour and adjacent tissues, as well as with conventional cell lines, and PROS1 immunohistochemistry with 15 paired tumour and adjacent tissues). Immune cell infiltration and tumour microenvironment, immune checkpoint inhibitors, immunomodulators, immune cell markers, and therapeutic outcomes in clinical studies of immune checkpoint blockade were subsequently explored, and we identified significant correlations with *PROS1* expression. Notably, a connection between *PROS1* expression and TAMs was revealed. Moreover, *PROS1* functions at the single-cell level, as well as the involvement of lncRNA-TF-gene triplets, were explored in LGG. Finally, *PROS1* expression was comprehensively analysed in combination with clinical diagnosis, treatment, and multi-omics data, including DNA methyltransferase expression and functional DNA methylation, expression of RNA modification and DNA mismatch repair system genes, copy number variation and single nucleotide variation frequency, *PROS1* alternative splicing, tumour genomic heterogeneity, and cancer stemness. These results suggest the involvement of PROS1 in glioma carcinogenesis. This conclusion is supported by a study indicating that PROS1 may play a vital role in the development of GBM by influencing cellular proliferation, migration, invasion, and apoptosis [11].

Because lncRNAs and TFs have been widely found to play indispensable roles in the occurrence and progression of tumours, the LncMAP database was used to explore TFs and lncRNAs that may regulate *PROS1* expression in LGG. Among seven TFs that may interact with *PROS1*, *NFKB1* was identified based on expression, survival, and correlation analyses. A previous study has shown that the lncRNA SLC26A4-AS1 promotes *NPTX1* transcriptional activity by recruiting *NFKB1*, thereby exerting antiangiogenic effects in glioma cells [22]. In the present study, we considered lncRNAs that were positively correlated with *PROS1* and *NFKB1*, but only lncRNA RP3-525N10.2 met all screening conditions, was significantly upregulated in LGG, and had the potential to improve the prognosis of patients with LGG. Taken together, our study findings suggest that lncRNA RP3-525N10.2 may decoy or guide *NFKB1*, thereby reducing *PROS1* expression and improving the poor prognosis in patients with LGG.

In view of the finite studies on *PROS1* functions, we annotated its functions using GO, KEGG pathways, and GESA. The results demonstrated that *PROS1*-related genes were focused on well-known processes, including T-cell receptor signalling, KEGG cancer pathways, transforming growth factor-β signalling, JAK-STAT signalling, and various other immune-related pathways. Functional analyses at the single-cell level showed that *PROS1* was upregulated in glioma cells, promoting cell invasion. Based on these results, we explored the relationships between *PROS1*-related genes and the prognosis of patients with LGG.

Immune cell infiltration in the tumour microenvironment and sufficient expression of immune checkpoint genes can alter the efficacy of chemotherapy, radiotherapy, or immunotherapy, thereby influencing the prognosis of patients with cancer. Our results demonstrated that *PROS1* expression was significantly positively correlated with the presence of most immune cells in LGG tissue, the expression of biomarkers by tumour-infiltrating immune cells, and the expression of immune checkpoint genes. Among these immune cells, TAMs are a rich cellular component of the brain TME that possess both tumour-promoting and immunosuppressive capacities [23]. Ubil et al. reported that PROS1 decreases M1 macrophage cytokine expression in vitro and in vivo [8]. Maimon et al. found that PROS1-deficient bone marrow-derived macrophages led to elevated TNF-α, IL-6, NOS2, and IL-10 levels via modulation of the SOCS3/NF-κB pathway [17]. Sadahiro et al. showed that *PROS1* is secreted by TAMs/microglia and subsequently physically associates with and activates AXL in mesenchymal glioma sphere cultures [20]. The TAM population can genetically be divided into at least two main groups: tissue-resident microglia/macrophages of embryonic origin and tissue-invading monocyte-derived macrophages [24, 25]. Their biomarkers extracted from CellMarker websites and the literature [26] are listed in Table 5, which shows that *PROS1* expression is significantly correlated with TAMs and might partially account for *PROS1*-mediated oncogenic mechanisms in LGG. Taken together, TAMs play a unique role in tumours, and LGG is no exception. Additionally, the significant association between immune checkpoint genes and *PROS1* indicated that targeting *PROS1* might increase the efficacy of immunotherapy in LGG, which was consistent with the Tumor Immune Dysfunction and Exclusion (TIDE) results. According to the DSigDB database, spiperone PC3, thapsigargin PC3, quercetin CTD 00006679, and pentabromodiphenyl ether CTD 00003077 were the best drug candidates.

This study has some limitations. First, the TCGA and CGGA databases inevitably neglect the inner tumour heterogeneity in different databases. Second, the possible mechanisms of DNMTs, MMRs, DNA methylation, alternative splicing, the identified lncRNA-TF-gene triplet and the roles of PROS1 in tumour migration, immune cell infiltration, and tumour escape should be explored in more detail in future studies. Third, although targeting PROS1 in patients with LGG is promising, a concern may be that PROS1 inhibits BBB breakdown, as it exerts a protective effect at the BBB [27].

**Fig. 15:**
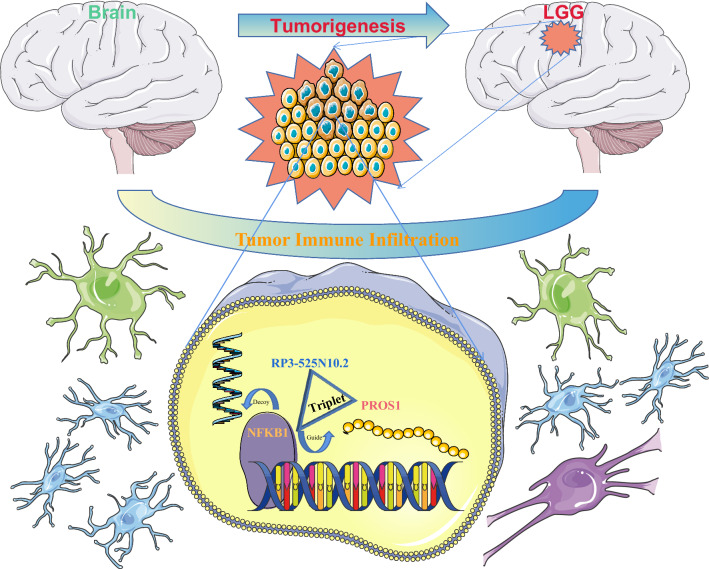
Graphical abstract of the model of RP3-525N10.2–NFKB1–PROS1 axis in carcinogenesis of LGG

In conclusion, this study elucidated that *PROS1* is upregulated in LGG and various other types of human cancers and may serve as a novel prognostic biomarker in LGG. PROS1 plays a vital pathogenic role in the immuno-oncological context of the TME, affects the infiltration of tumour tissue by immune cells, and influences patient prognosis. Poor prognosis is associated with epigenetic modifications, genomic heterogeneity, cancer stemness, and alternative splicing of *PROS1*. Moreover, we identified a possible regulatory mechanism of *PROS1* in LGG, namely the lncRNA RP3-525N10.2-*NFKB1*-*PROS1* triplet (Fig. 15). Collectively, our study suggests that PROS1 could serve as a biomarker for cancer diagnosis, prognosis, therapy selection, and follow-up.

## Supplementary Information

**Additional file1: Table S1.** The association between PROS1 expression and clinicopathological characteristic. **Table S2. **PROS1 expression association with clinical pathological characteristics (logistic regression). **Table S3.** Univariate regression and multivariate survival method (Overall Survival) of prognostic covariates LGG patients. **Figure S1.** *PROS1 *expression in 31 types of tissues using the GTEx dataset. **Figure S2.** *PROS1 *expression in 21 tumour cell lines using the Cancer Cell Line Encyclopedia database. **Figure S3. PROS1 **expression between tumour and normal tissues using The Cancer Genome Atlas (TCGA) database. **Figure S4.** PROS1 expression is associated with positive result of genomic heterogeneity and cancer stemness. **Figure S5. **PROS1 expression is associated with negative result of genomic heterogeneity and cancer stemness. **Figure S6.** Correlation between PROS1 expression and clinical parameters of patients with LGG. **Figure S7.** Correlation between PROS1 expression and disease-specific survival in 33 tumours from the TCGA database. **Figure S8.** Correlation between PROS1 expression and disease-free interval in 33 tumours from the TCGA database. **Figure S9.** Correlation between PROS1 expression and progression-free interval in 33 tumours from the TCGA database. **Figure S10.** Single-cell analysis of PROS1 functions in patients with LGG. **Figure S11.** Correlation between PROS1 expression and TAMs immune cell markers in LGG.

## Data Availability

All data are available on public repositories, which are listed in the main context.

## Abbreviations

TCGA: The Cancer Genome Atlas
CGGA: Chinese Glioma Genome Atlas
CCK-8: Cell counting kit-8
EdU: 5-Ethynyl-2’-deoxyuridine
PROS1: Protein S1
CCLE: Cancer cell line encyclopedia database
GEPIA: Gene Expression Profiling Interactive Analysis
TIMER: Tumor Immune Estimation Resource
MSI: Microsatellite instability
NEO: Neoantigen
DMPss: Differentially methylated probes-based stemness
EREG.EXPss: Epigenetically regulated RNA expression-based stemness
TIME: Tumor immune microenvironment
ICI: Immune checkpoint inhibitor
RNA-seq: RNA sequencing
OS: Overall survival
GTEx: Genotype-tissue expression
FPKM: Fragments per kilobase of exon model per million mapped reads
DEGs: Differentially expressed genes
GO: Gene ontology
KEGG: Kyoto encyclopedia of genes and genomes
NMF: Non-negative matrix factorization
tSNE: T-distributed stochastic neighbor embedding
K-M: Kaplan–Meier
ssGSEA: Single sample gene set enrichment analysis
TMB: Tumor mutation burden
CNA: Copy number alteration
TIDE: Tumor immune dysfunction and exclusion
MSI: Microsatellite instability
ROC: Receiver operating characteristic
HR: Hazard ratio
qRT-PCR: Quantitative real-time polymerase chain reaction
WHO: World Health Organization
ACC: Adrenocortical carcinoma
BLCA: Bladder urothelial carcinoma
BRCA: Breast invasive carcinoma
CESC: Cervical squamous cell carcinoma and endocervical adenocarcinoma
CHOL: Cholangiocarcinoma
COAD: Colon adenocarcinoma
COADREAD: Colon adenocarcinoma/Rectum adenocarcinoma Esophageal carcinoma
DLBC: Lymphoid Neoplasm Diffuse Large B-cell Lymphoma
ESCA: Esophageal carcinoma
GBM: Glioblastoma multiforme
GBMLGG: Glioma
HNSC: Head and Neck squamous cell carcinoma
KICH: Kidney Chromophobe
KIPAN: Pan-kidney cohort (KICH + KIRC + KIRP)
KIRC: Kidney renal clear cell carcinoma
KIRP: Kidney renal papillary cell carcinoma
LAML: Acute Myeloid Leukemia
LIHC: Liver hepatocellular carcinoma
LUAD: Lung adenocarcinoma
LUSC: Lung squamous cell carcinoma
MESO: Mesothelioma
OV: Ovarian serous cystadenocarcinoma
PAAD: Pancreatic adenocarcinoma
PCPG: Pheochromocytoma and Paraganglioma
PRAD: Prostate adenocarcinoma
READ: Rectum adenocarcinoma
SARC: Sarcoma
STAD: Stomach adenocarcinoma
SKCM: Skin cutaneous melanoma
STES: Stomach and esophageal carcinoma
TGCT: Testicular Germ Cell Tumors
THCA: Thyroid carcinoma
THYM: Thymoma
UCEC: Uterine corpus endometrial carcinoma
UCS: Uterine carcinosarcoma
UVM: Uveal melanoma
OS: Osteosarcoma
