# Supplementary material for: LncRNA RP3-525N10.2-NFKB1-PROS1 triplet-mediated low PROS1 expression is an onco-immunological biomarker in low-grade gliomas: a pan-cancer analysis with experimental verification
Source: J Transl Med. 2022 Jul 25;20:335. doi: 10.1186/s12967-022-03536-y (PMC9310492; doi:10.1186/s12967-022-03536-y)
Supplement: Supplementary file 1 — Additional file1: Table S1. The association between PROS1 expression and clinicopathological characteristic. Table S2. PROS1 expression association with clinical pathological characteristics (logistic regression). Table S3. Univariate regression and multivariate survival method (Overall Survival) of prognostic covariates LGG patients. Figure S1. PROS1 expression in 31 types of tissues using the GTEx dataset. Figure S2. PROS1 expression in 21 tumour cell lines using the Cancer Cell Line Encyclopedia database. Figure S3. PROS1 expression between tumour and normal tissues using The Cancer Genome Atlas (TCGA) database. Figure S4. PROS1 expression is associated with positive result of genomic heterogeneity and cancer stemness. Figure S5. PROS1 expression is associated with negative result of genomic heterogeneity and cancer stemness. Figure S6. Correlation between PROS1 expression and clinical parameters of patients with LGG. Figure S7. Correlation between PROS1 expression and disease-specific survival in 33 tumours from the TCGA database. Figure S8. Correlation between PROS1 expression and disease-free interval in 33 tumours from the TCGA database. Figure S9. Correlation between PROS1 expression and progression-free interval in 33 tumours from the TCGA database. Figure S10. Single-cell analysis of PROS1 functions in patients with LGG. Figure S11. Correlation between PROS1 expression and TAMs immune cell markers in LGG. [file 12967_2022_3536_MOESM1_ESM.pdf]

Table S1. The association between PROS1 expression and clinicopathological characteristic

Characteristic	levels	Low expression of PROS1	High expression of PROS1	p	Test
n		255	255		
WHO grade, n (%)	G2	125 (27.6%)	91 (20.1%)	**7.1e-07**	Chisq.test
	G3	101 (22.3%)	136 (30%)		
IDH status, n (%)	WT	33 (6.5%)	61 (12%)	**7.2e-06**	Chisq.test
	Mut	220 (43.4%)	193 (38.1%)		
1p/19q codeletion, n (%)	codel	88 (17.3%)	80 (15.7%)	0.15	Chisq.test
	non-codel	167 (32.7%)	175 (34.3%)		
Primary therapy outcome, n (%)	PD	42 (9.5%)	59 (13.4%)	0.03	Chisq.test
	SD	73 (16.6%)	70 (15.9%)		
	PR	30 (6.8%)	32 (7.3%)		
	CR	78 (17.7%)	56 (12.7%)		
Gender, n (%)	Female	111 (21.8%)	117 (22.9%)	0.656	Chisq.test
	Male	144 (28.2%)	138 (27.1%)		
Race, n (%)	Asian	4 (0.8%)	4 (0.8%)	1.000	Fisher.test
	Black or African American	11 (2.2%)	10 (2%)		
	White	238 (47.7%)	232 (46.5%)		
Age, n (%)	<=40	140 (27.5%)	112 (22%)	**0.067**	Chisq.test
	>40	115 (22.5%)	143 (28%)		
Histological type, n (%)	Astrocytoma	83 (16.3%)	109 (21.4%)	0.02	Chisq.test
	Oligoastrocytoma	68 (13.3%)	60 (11.8%)		

Characteristic	levels	Low expression of PROS1	High expression of PROS1	p	Test
	Oligodendroglioma	104 (20.4%)	86 (16.9%)		
Laterality, n (%)	Left	134 (26.5%)	114 (22.6%)	0.144	Fisher.test
	Midline	2 (0.4%)	4 (0.8%)		
	Right	116 (23%)	135 (26.7%)		
OS event, n (%)	Alive	205 (40.2%)	180 (35.3%)	**2.6e-03**	Chisq.test
	Dead	50 (9.8%)	75 (14.7%)		
DSS event, n (%)	Alive	207 (41.2%)	182 (36.3%)	**3e-03**	Chisq.test
	Dead	44 (8.8%)	69 (13.7%)		
PFI event, n (%)	Alive	166 (32.5%)	152 (29.8%)	0.03	Chisq.test
	Dead	89 (17.5%)	103 (20.2%)		
Age, meidan (IQR)		39 (31, 52)	43 (33, 54)	**0.026**	Wilcoxon

Table S2. PROS1 expression association with clinical pathological characteristics (logistic regression).

Characteristics	Total(N)	Odds Ratio(OR)	P value
WHO grade (G3 vs. G2)	453	**1.850 (1.276-2.692)**	**0.001**
1p/19q codeletion (non-codel vs. codel)	510	1.153 (0.797-1.670)	0.451
IDH status (Mut vs. WT)	507	**0.475 (0.295-0.751)**	**0.002**
Gender (Male vs. Female)	510	0.909 (0.641-1.289)	0.593
Age (>40 vs. <=40)	510	**1.554 (1.097-2.207)**	**0.013**
Histological type (Oligoastrocytoma&Oligodendroglioma vs. Astrocytoma)	510	**0.646 (0.450-0.926)**	**0.018**
Laterality (Midline&Right vs. Left)	505	1.385 (0.976-1.967)	0.068
Race (Black or African American&Asian vs. White)	499	0.957 (0.447-2.037)	0.910
Primary therapy outcome (PR&CR vs. SD&PD)	440	0.726 (0.497-1.059)	0.097

Table S3. Univariate regression and multivariate survival method (Overall Survival) of prognostic covariates LGG patients

Characteristics	Total(N)	Univariate analysis		Multivariate analysis	
		Hazard ratio (95% CI)	P value	Hazard ratio (95% CI)	P value
PROS1	509	1.548 (1.260-1.903)	**<0.001**	1.297 (1.028-1.636)	**0.028**
WHO grade(G2 vs G3)	452	3.059 (2.046-4.573)	**<0.001**	2.231 (1.385-3.595)	**<0.001**
Age(<=40 vs >40)	509	2.889 (2.009-4.155)	**<0.001**	2.755 (1.834-4.139)	**<0.001**
Gender（Female vs Male）	509	1.124 (0.800-1.580)	0.499	1.013 (0.736–1.350)	0.579
IDH status(Mut vs WT)	506	5.385 (3.777-7.679)	**<0.001**		
1p/19q codeletion(non-codel vs codel)	509	2.493 (1.590-3.910)	**<0.001**		
Primary therapy outcome(PR&CR vs PD&SD)	439	4.963 (2.782-8.851)	**<0.001**		
Race(Black or African American&Asian vs White)	498	1.178 (0.549-2.529)	0.675		
Histological type(Astrocytoma vs Oligoastrocytoma&Oligodendroglioma)	509	0.606 (0.430-0.853)	**0.004**		
Laterality(Left& Right vs Midline)	504	1.203 (0.372-3.886)	0.758		

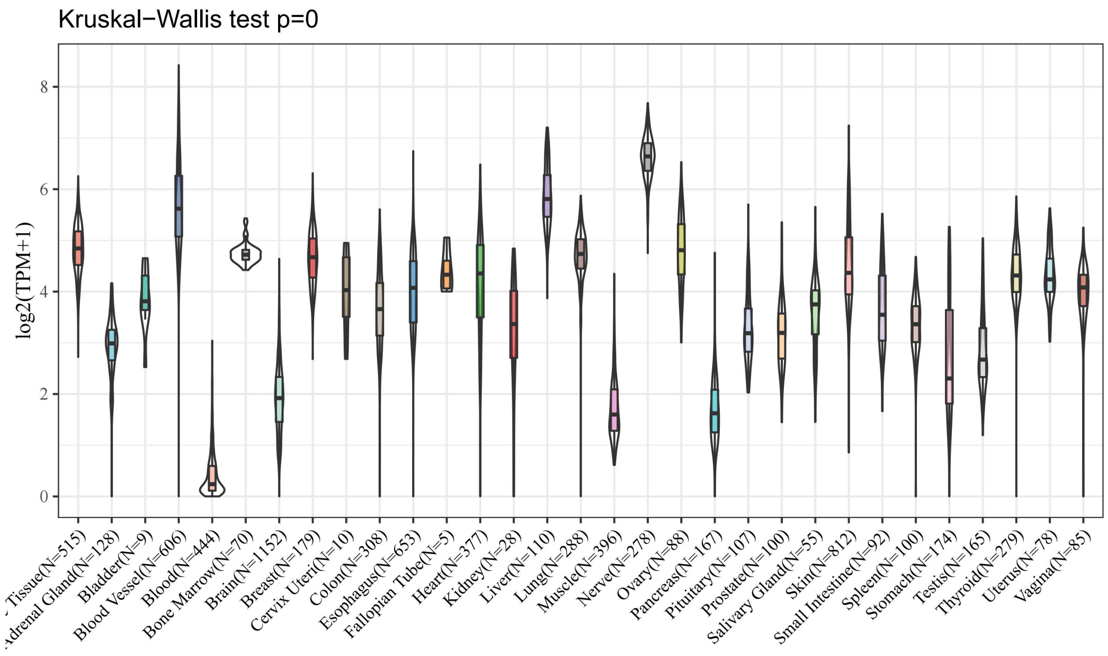

Figure S1. *PROS1* expression in 31 types of tissues using the GTEx dataset

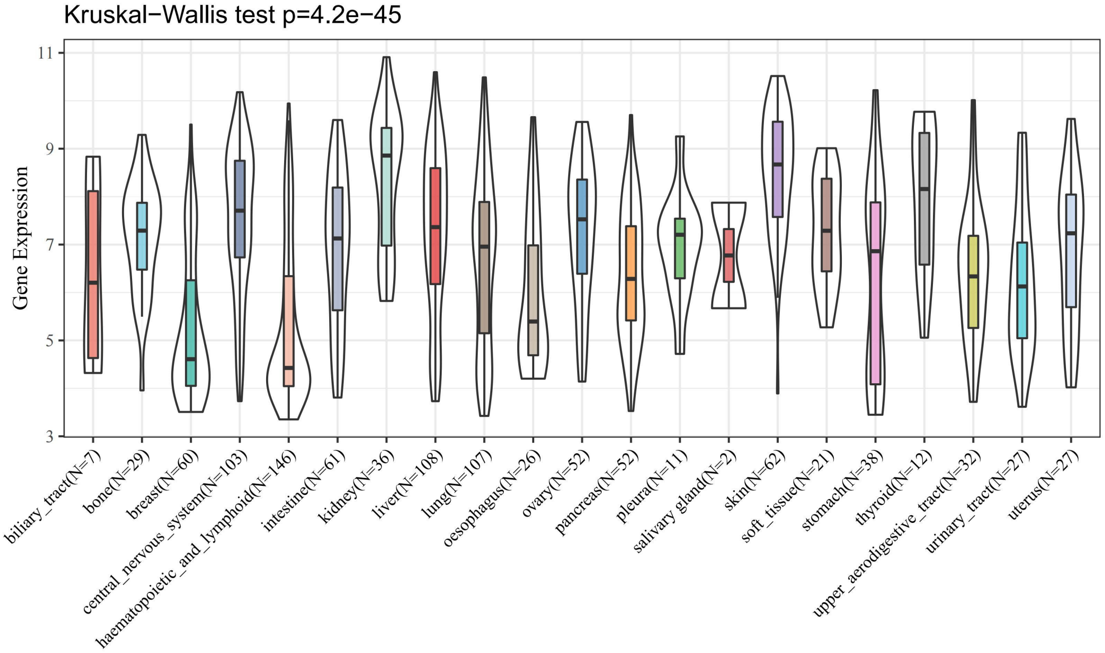

Figure S2. *PROS1* expression in 21 tumour cell lines using the Cancer Cell Line Encyclopedia database

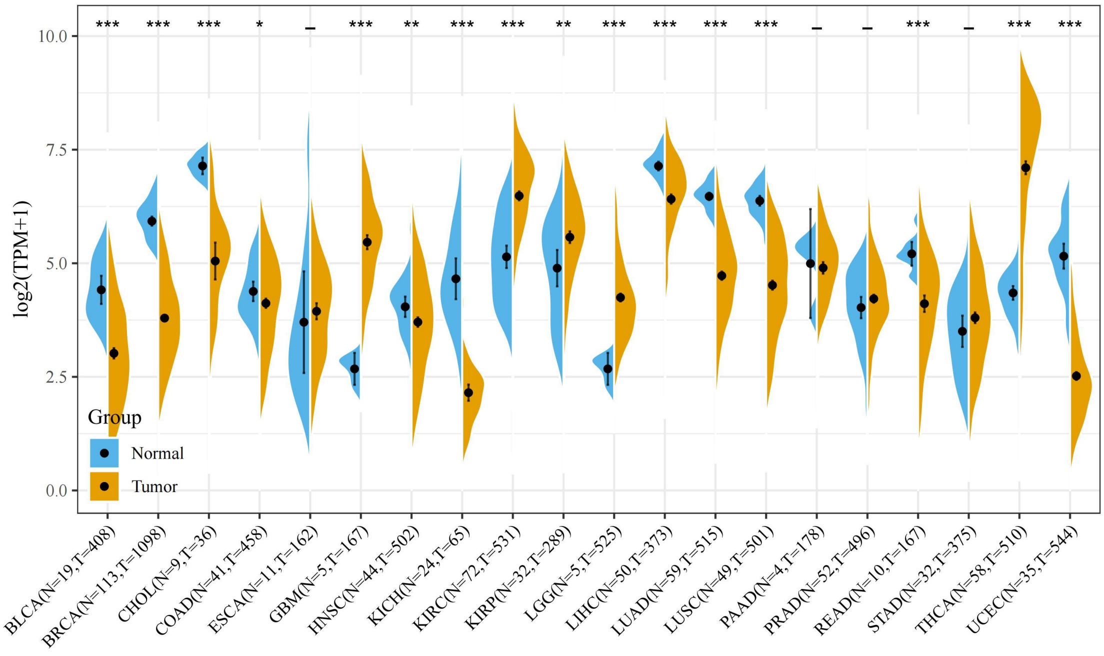

Figure S3. *PROS1* expression between tumour and normal tissues using The Cancer Genome Atlas (TCGA) database

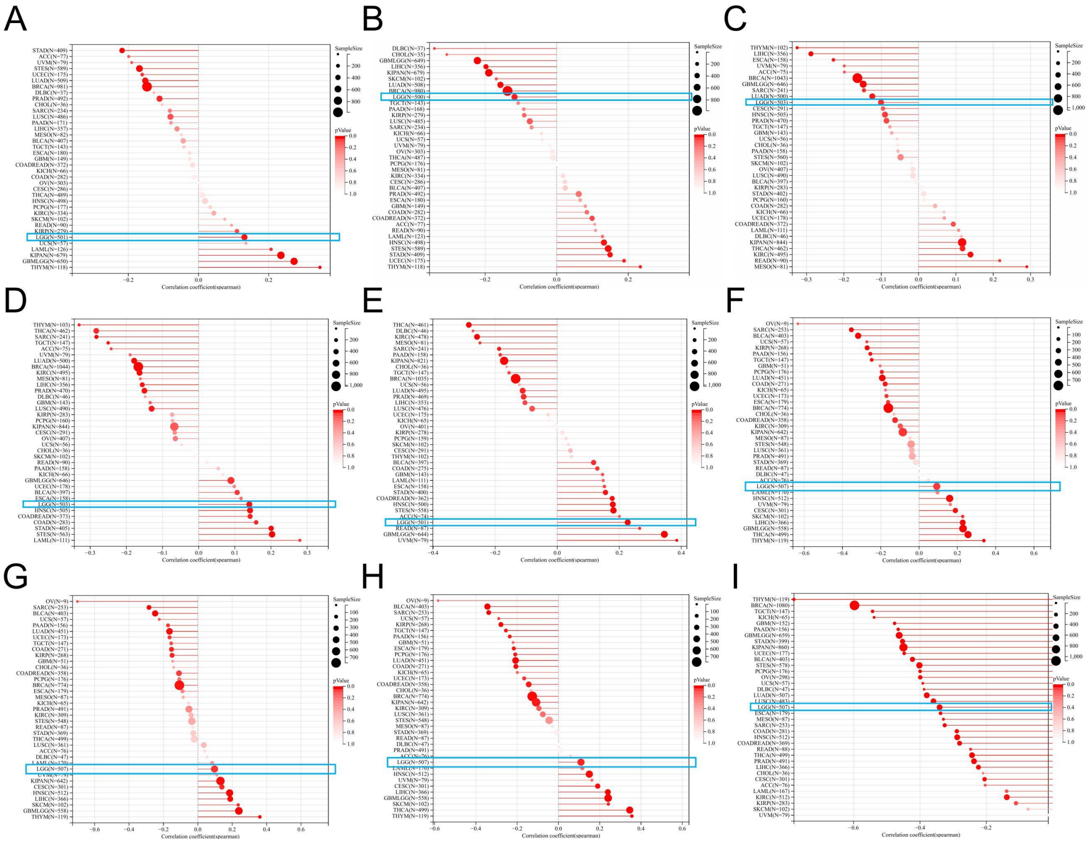

Figure S4. PROS1 expression is associated with positive result of genomic heterogeneity and cancer stemness. (A) tumour mutational burden (TMB), (B) mutant-allele tumour heterogeneity (MATH), (C) tumour ploidy, (D) homologous recombination deficiency (HRD), (E) loss of heterozygosity (LOH), (F) DNA

methylation-based stemness (DNAss), (G) enhancer elements/DNA methylation-based stemness (ENHss), (H) epigenetically regulated DNA methylation-based stemness (EREG-METHss), and (I) RNA expression-based stemness (RNAss).

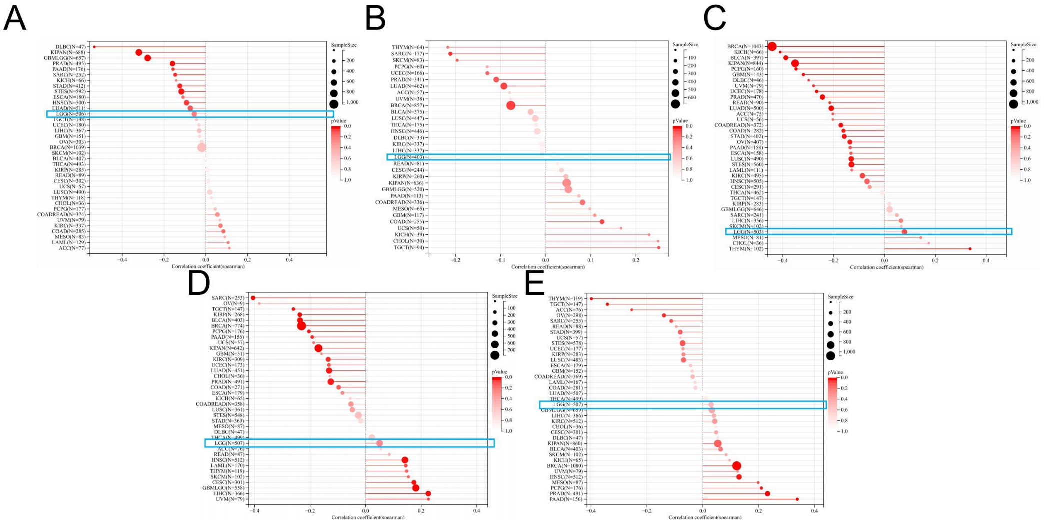

Figure S5. PROS1 expression is associated with negative result of genomic heterogeneity and cancer stemness. (A) microsatellite instability (MSI), (B) neoantigen (NEO), (C) tumour purity, (D) differentially methylated probes-based stemness (DMPss), and (E) epigenetically regulated RNA expression-based stemness (EREG.EXPss).

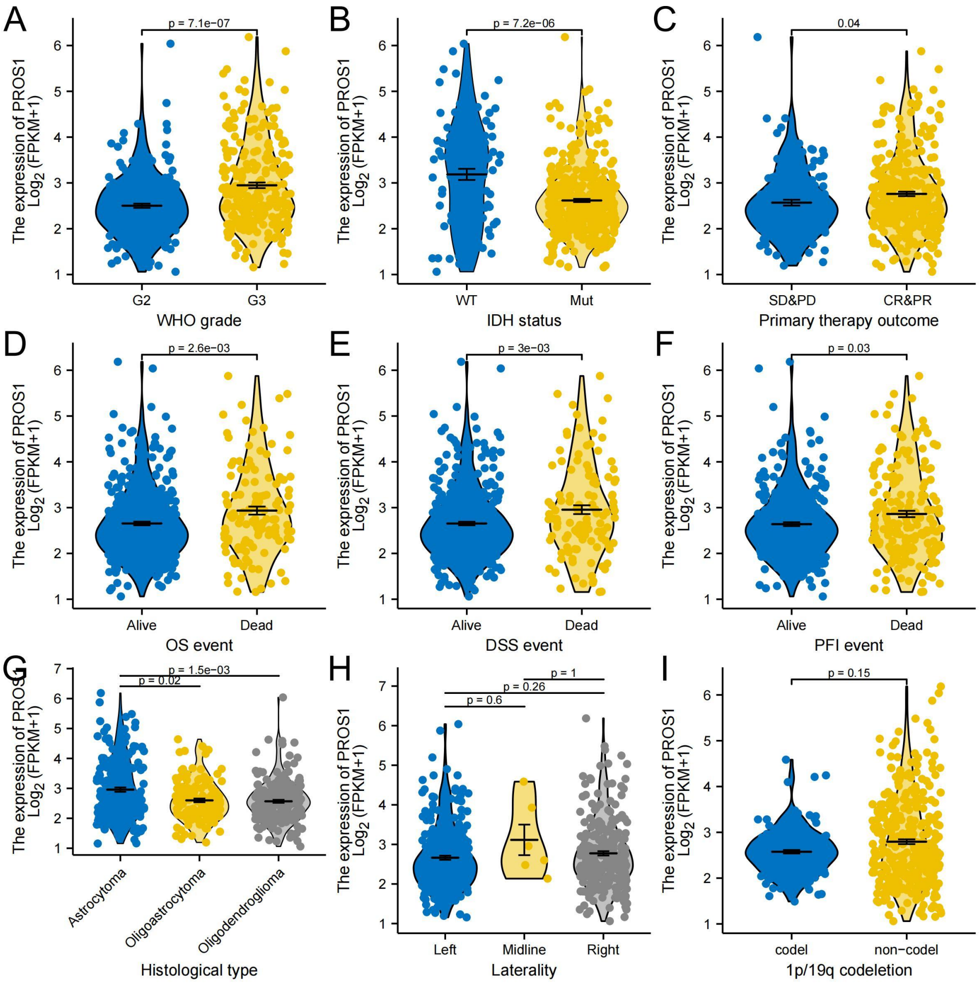

Figure S6. Correlation between PROS1 expression and clinical parameters of patients with LGG. (A) WHO grade, (B) IDH status, (C) primary therapy outcome, (D) overall survival, (E) disease-specific survival, (F) progression-free interval (G) histological type, (H) Laterality, and (I) histological type,1p/19q codeletion.

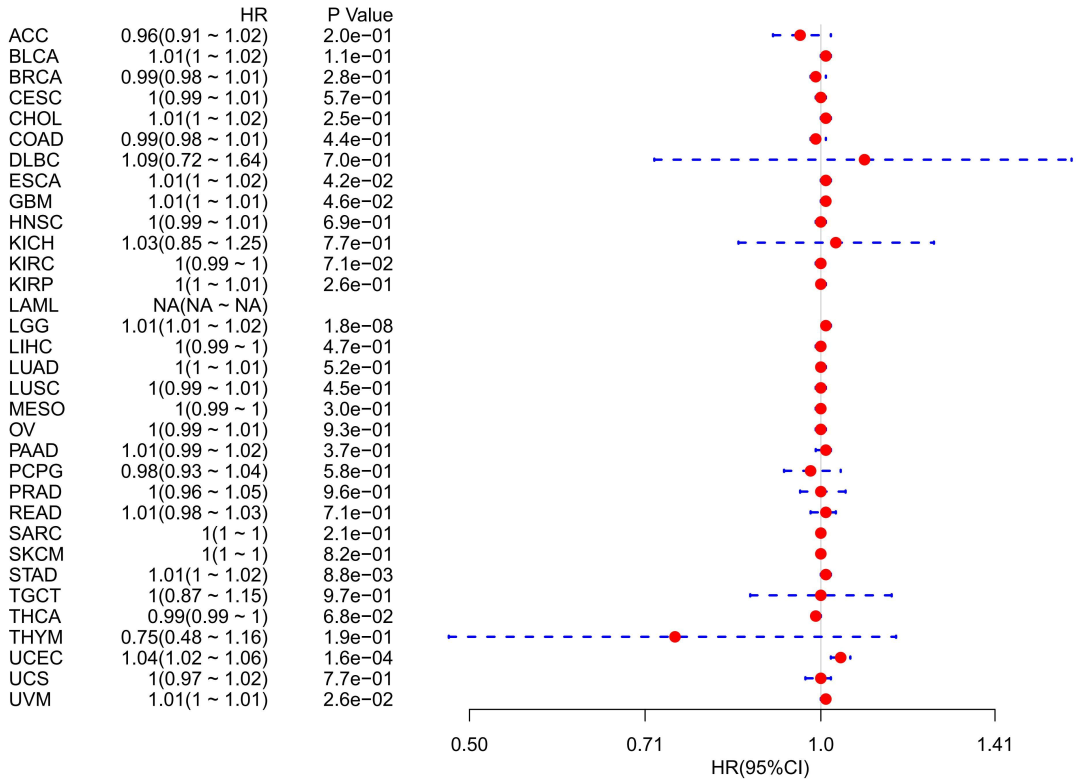

Figure S7. Correlation between PROS1 expression and disease-specific survival in 33 tumours from the TCGA database.

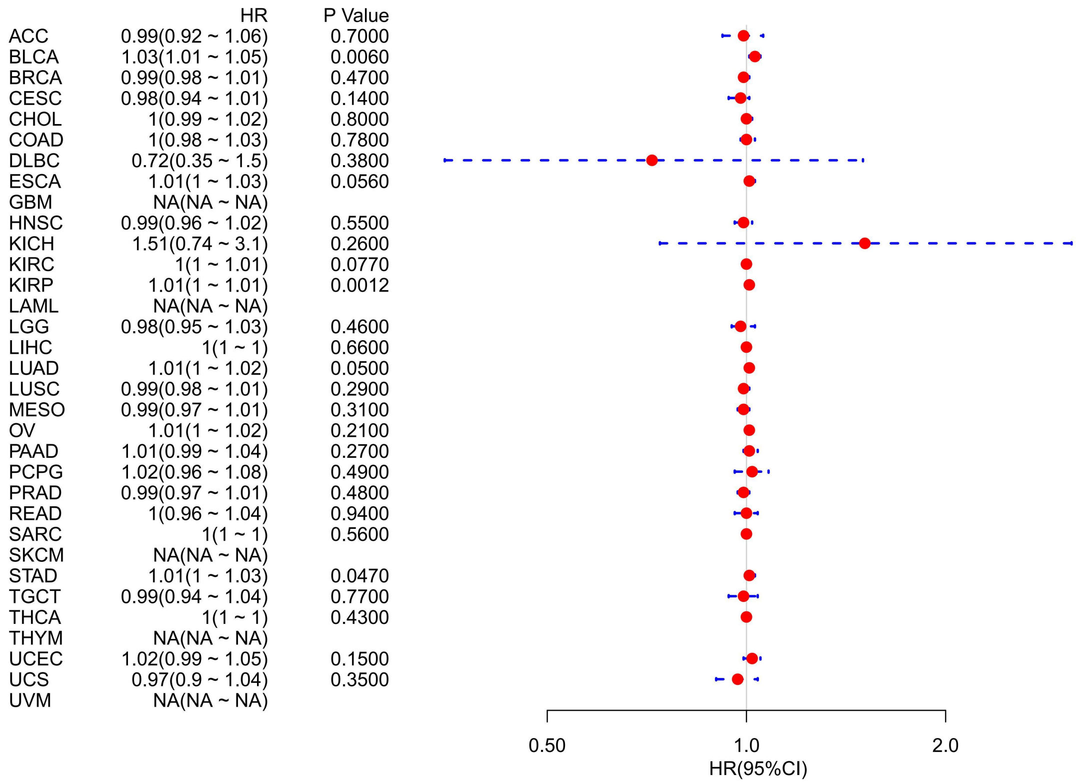

Figure S8. Correlation between PROS1 expression and disease-free interval in 33 tumours from the TCGA database.

	HR	P Value
ACC	0.99(0.96 ~ 1.03)	7.1e−01
BLCA	1.02(1.01 ~ 1.03)	1.4e−03
BRCA	0.99(0.98 ~ 1.01)	2.8e−01
CESC	1(0.99 ~ 1.01)	8.7e−01
CHOL	1(0.99 ~ 1.01)	4.4e−01
COAD	1(0.99 ~ 1.01)	8.9e−01
DLBC	0.97(0.74 ~ 1.28)	8.5e−01
ESCA	1.01(1 ~ 1.02)	7.4e−02
GBM	1.01(1 ~ 1.01)	1.2e−02
HNSC	1(0.99 ~ 1.01)	5.6e−01
KICH	1.03(0.89 ~ 1.2)	6.6e−01
KIRC	1(1 ~ 1)	6.1e−01
KIRP	1.01(1 ~ 1.01)	8.3e−03
LAML	NA(NA ~ NA)	
LGG	1.01(1.01 ~ 1.01)	2.6e−07
LIHC	1(1 ~ 1)	7.3e−01
LUAD	1.01(1 ~ 1.01)	3.0e−03
LUSC	1(0.99 ~ 1)	2.4e−01
MESO	1(0.99 ~ 1)	1.8e−01
OV	1.01(1 ~ 1.02)	2.2e−01
PAAD	1(0.99 ~ 1.01)	5.0e−01
PCPG	1.02(0.99 ~ 1.04)	1.6e−01
PRAD	0.99(0.97 ~ 1)	6.0e−02
READ	1.01(0.99 ~ 1.02)	4.2e−01
SARC	1(1 ~ 1)	3.0e−01
SKCM	1(1 ~ 1)	6.0e−01
STAD	1.01(1.01 ~ 1.02)	9.3e−04
TGCT	1.01(0.96 ~ 1.05)	7.7e−01
THCA	1(1 ~ 1)	6.0e−01
THYM	1.02(0.99 ~ 1.05)	2.9e−01
UCEC	1.02(1 ~ 1.04)	3.3e−02
UCS	0.99(0.97 ~ 1.02)	5.2e−01
UVM	1.01(1 ~ 1.01)	5.2e−03

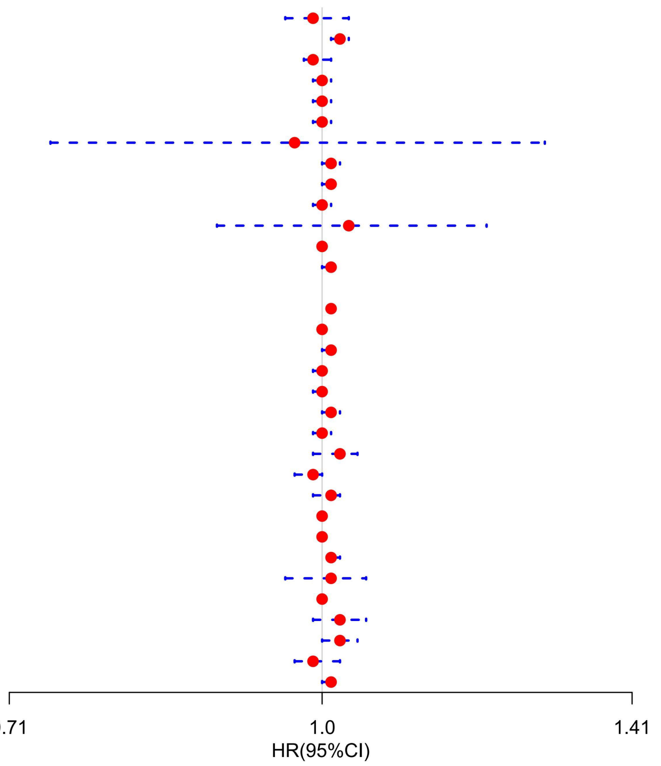

Figure S9. Correlation between PROS1 expression and progression-free interval in 33 tumours from the TCGA database.

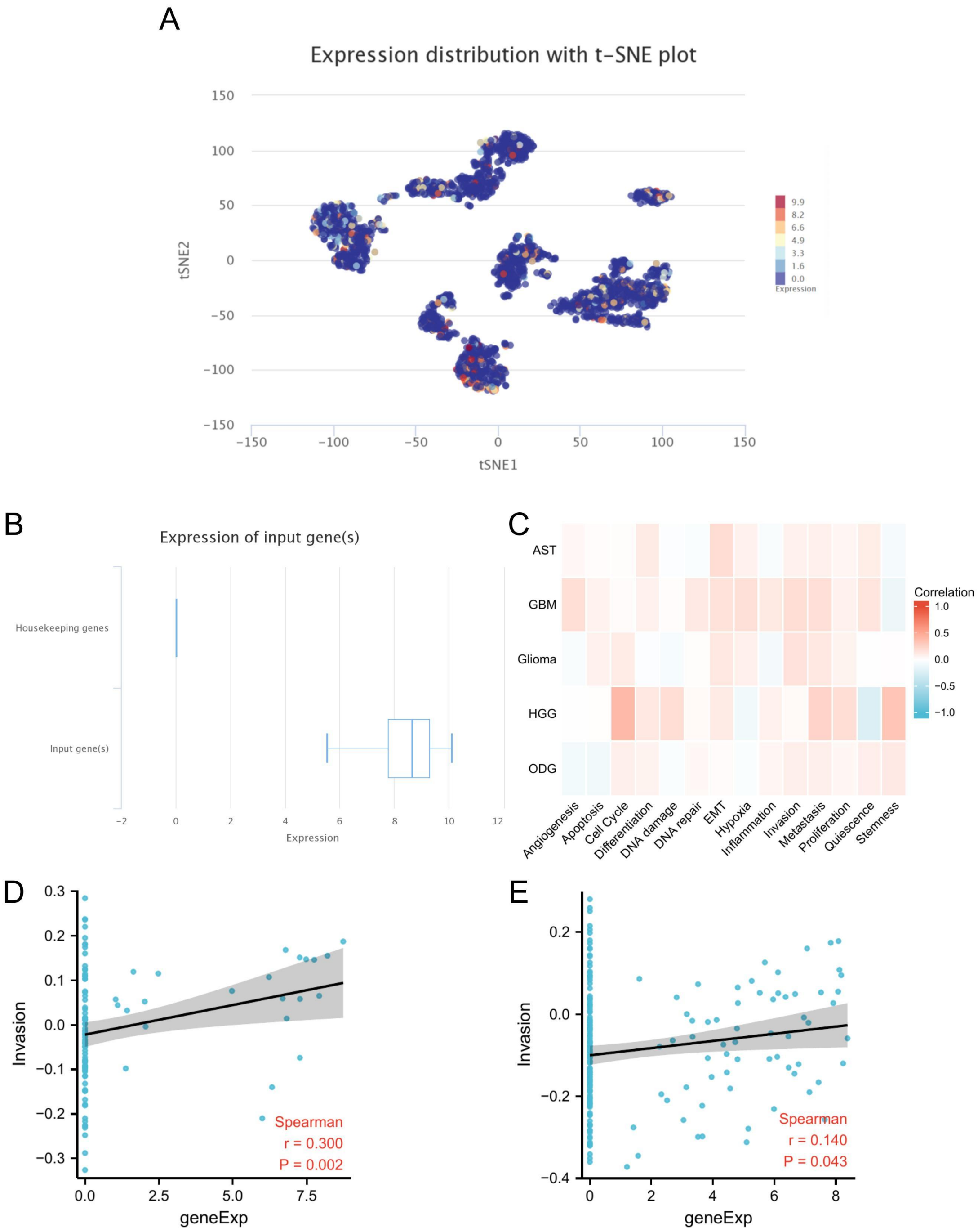

Figure S10. Single-cell analysis of PROS1 functions in patients with LGG.

A

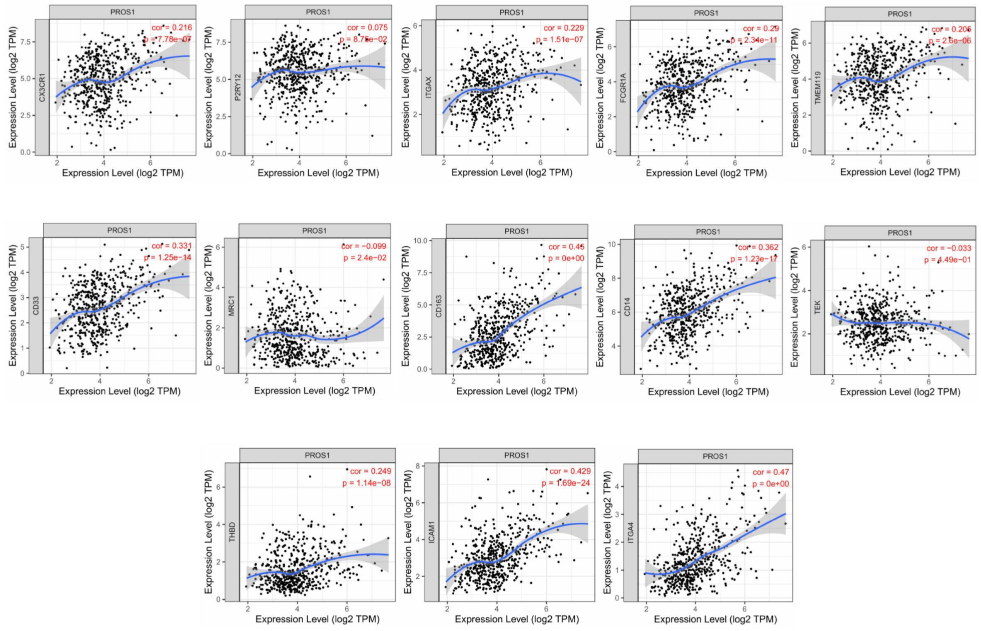

B

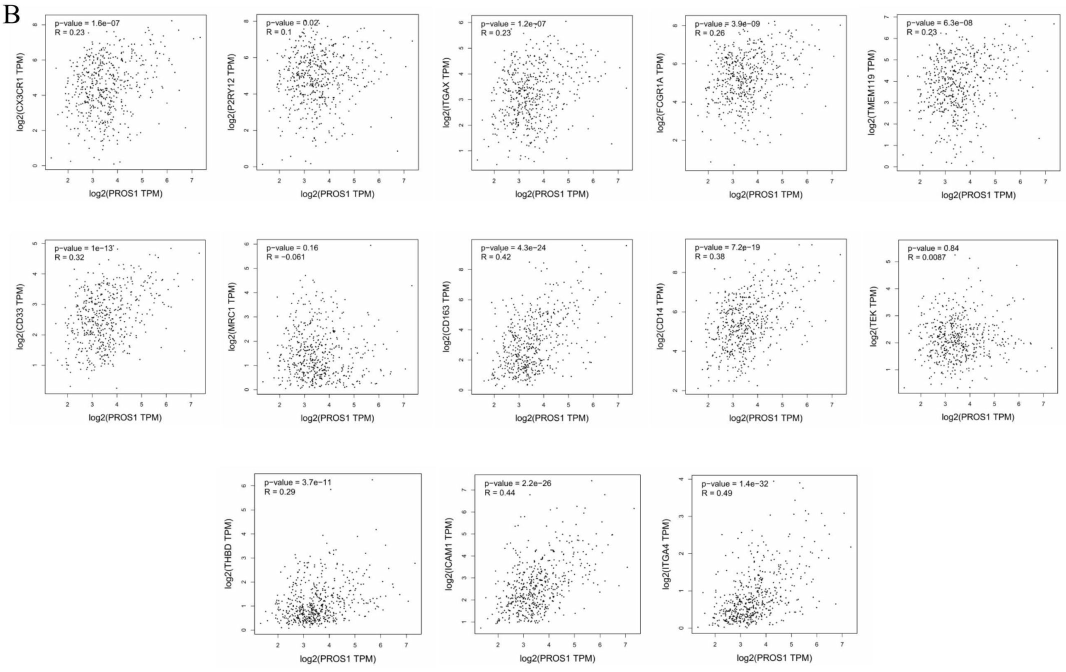

Figure S11. Correlation between PROS1 expression and TAMs immune cell markers in LGG.
